# Water Flow in Single-Wall Nanotubes: Oxygen Makes It Slip, Hydrogen Makes It Stick

**DOI:** 10.1021/acsnano.2c02784

**Published:** 2022-06-21

**Authors:** Fabian L. Thiemann, Christoph Schran, Patrick Rowe, Erich A. Müller, Angelos Michaelides

**Affiliations:** † Thomas Young Centre, London Centre for Nanotechnology and Department of Physics and Astronomy, University College London, Gower Street, London WC1E 6BT, United Kingdom; ‡ Yusuf Hamied Department of Chemistry, University of Cambridge, Lensfield Road, Cambridge CB2 1EW, United Kingdom; § Department of Chemical Engineering, Sargent Centre for Process Systems Engineering, Imperial College London, South Kensington Campus, London SW7 2AZ, United Kingdom

**Keywords:** nanofluidics, liquid/solid friction, nanotubes, confined water, machine learning potentials, carbon, boron nitride

## Abstract

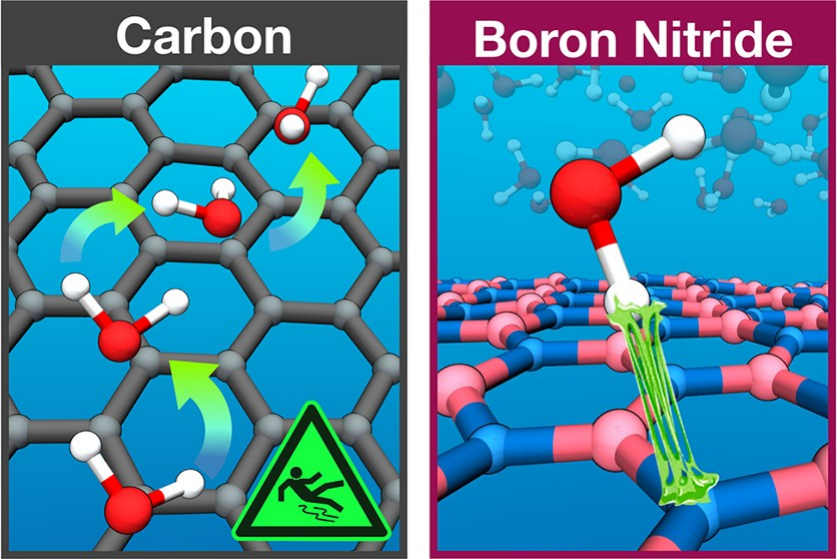

Experimental measurements have reported ultrafast and radius-dependent water transport in carbon nanotubes which are absent in boron nitride nanotubes. Despite considerable effort, the origin of this contrasting (and fascinating) behavior is not understood. Here, with the aid of machine learning-based molecular dynamics simulations that deliver first-principles accuracy, we investigate water transport in single-wall carbon and boron nitride nanotubes. Our simulations reveal a large, radius-dependent hydrodynamic slippage on both materials, with water experiencing indeed a ≈5 times lower friction on carbon surfaces compared to boron nitride. Analysis of the diffusion mechanisms across the two materials reveals that the fast water transport on carbon is governed by facile oxygen motion, whereas the higher friction on boron nitride arises from specific hydrogen–nitrogen interactions. This work not only delivers a clear reference of quantum mechanical accuracy for water flow in single-wall nanotubes but also provides detailed mechanistic insight into its radius and material dependence for future technological application.

The ability of water to flow seemingly friction-less across graphitic surfaces^1−7^ has put carbon nanotubes (CNTs) at the forefront of nanofluidic^8,9^ applications in the fields of desalination,^10,11^ water filtration,^12,13^ and blue energy harvesting.^14^ In particular, recent experiments^3^ in CNTs have shown that water exhibits an enormous and curvature-dependent hydrodynamic slippage (low friction) with smaller radii resulting in a greater slippage. In contrast, in isostructural but electronically different boron nitride nanotubes (BNNTs), no slip was detected. To exploit the full potential of low-dimensional materials for nanofluidic devices, a clear understanding of the physical mechanisms behind this radius and material dependence is required.^15^

Despite more than a decade of intense research, however, our understanding of the transport properties of water inside nanotubes remains far from complete. This lack of insight partially arises from (i) differences in the systems studied experimentally (single multiwalled CNTs,^16^ carbon nanoconduits,^4−6^ and membranes of aligned CNTs^1,2^); (ii) the challenge of accurately measuring flow through extremely narrow channels; and (iii) the likely sensitivity of the results to impurities and defects that are inevitably present. Molecular dynamics (MD) simulations allow, in principle, for these challenges to be bypassed.^17^ However, when classical MD simulations have been performed, the results obtained are highly sensitive to the interaction models used and computational setups employed, showing a 3 orders of magnitude spread for the flow enhancement of water inside CNTs.^18^ In addition, classical MD simulations fail to explain the experimentally observed radius dependence in the diameter range between ≈30 and 100 nm.^19,20^ Ab initio MD (AIMD), conversely, could provide the required accuracy by accounting explicitly for the electronic structure of the systems studied.^21,22^ Indeed AIMD simulations have revealed that water exhibits a 3–5 times larger friction on hexagonal boron nitride surfaces compared to graphene.^23,24^ These studies, however, have been limited to flat sheets, as the high computational cost of AIMD impedes the simulation of large diameter nanotubes. The inherent constraints on the accessible length and time scales, moreover, inevitably introduce finite size errors and question marks over the convergence of the dynamical quantities computed. Thus, despite the progress made, a systematic study of both the radius and material dependence—using techniques that accurately tackle the interatomic interactions and dynamical properties—has yet to be performed.

Here, we rise to this challenge and report the findings of a detailed first-principles machine learning (ML)-based MD study of water transport in single-wall CNTs and BNNTs. The key aims of this work are (i) to obtain reliable reference quality first-principles values for water flow and in so-doing shed light on the myriad of simulation results in the literature;^18^ and (ii) to gain molecular-level understanding of the mechanisms of water transport in low-dimensional materials. By reliably representing the potential energy surface (PES) of a chosen first-principles reference method, machine learning potentials (MLPs) have become a powerful approach for simulating complex systems, achieving quantum mechanical accuracy at a fraction of the usual cost and, thus, facilitating simulations at longer time and length scales.^25−28^ Employing our recently introduced methodology for the rapid development of MLPs^29^ allows us to do precisely this, thereby achieving converged statistics while maintaining first-principles accuracy. A detailed overview of this approach can be found in the Methodology section, section S1.B of the Supporting Information, and the original reference.^29^ The simulation lengths and systems sizes of this work go beyond previous AIMD studies by at least an order of magnitude with more than 40 ns of high-quality simulation data obtained on nanotubes varying in diameter between ≈1.6 and ≈5.5 nm. This allows to provide a clear reference of quantum mechanical accuracy for water flow in single-wall nanotubes.

In agreement with experiments^6^ and previous AIMD studies,^23,24^ we find that water indeed experiences a significantly larger friction in BNNTs compared to CNTs. The strong curvature dependence, conversely, is by no means unique to the water–carbon couple but also occurs in BNNTs. Beyond providing a firm theoretical foundation for flow through pristine single-wall nanotubes, our simulations allow insight into the elementary processes involved. Specifically, we find that the differences between the two materials originate from alternating docking and hopping events induced by the hydrogen–nitrogen interaction only present in BNNTs (hydrogen imposed). The radius dependence observed, conversely, is mainly of geometric nature where a higher curvature results in a smoother free energy landscape, that is, lower energy barriers, and, thus, smaller friction (oxygen imposed). Having a clear understanding of these mechanisms is expected to be of great importance for materials design of nanofluidic devices, suggesting routes for directional flow via tailor-made nanotubes or two-dimensional nanostructures. In this way, our work pushes forward our understanding of water transport under confinement and helps to close a long-standing knowledge gap^15^ in the field of nanofluidics.

## Results and Discussion

### Determination of the Material and Radius-Dependent Friction Based on First-Principles Quality Machine Learning Potentials

**Figure 1:**
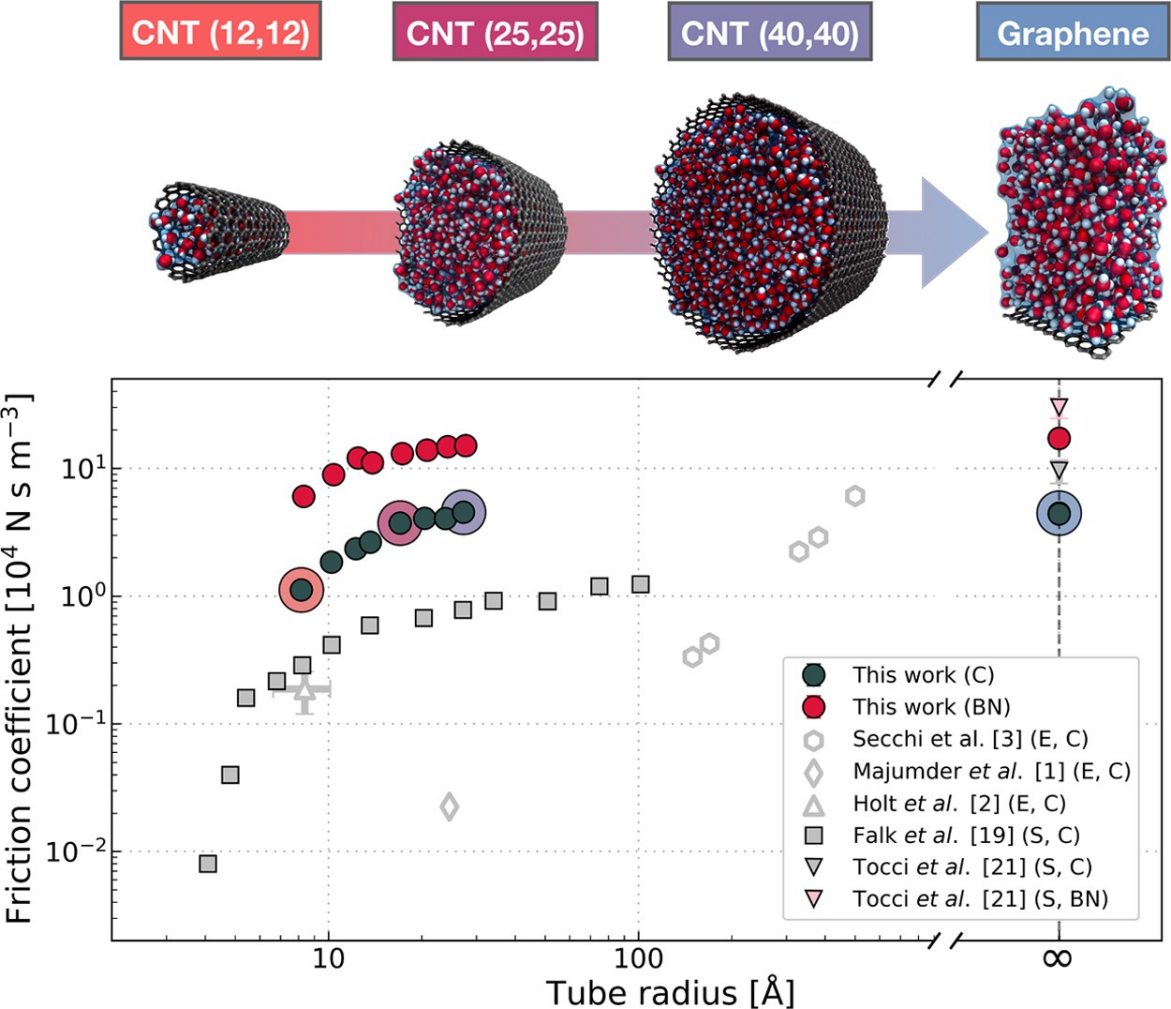
Friction of water inside CNTs and BNNTs of different diameters. The top panel shows snapshots of the simulations of the selected CNTs and graphene with increasing diameter from left to right. In the bottom panel, we report the friction coefficient as a function of tube radius showing our results as well as a small selection of previous experimental and computational work. Depending on the type of study, the related data are labeled with E (experiment) and S (simulation), respectively. Similarly, the confining material investigated is indicated by C (CNTs and graphene) and BN (BNNTs and hBN). The circles around the data points in the lower panel correspond to the systems shown in the top panel with the corresponding color. From our simulations, the statistical error was obtained from splitting the trajectory into two blocks; however, the magnitude of the error is small compared to the marker size on the log–log scale.

Using the approach introduced in ref (29), we developed and validated MLPs to probe the systems targeted in this study. Details of the approach used and validations are provided in the Methodology section and the Supporting Information. With these MLPs, we proceed to benchmark the hydrodynamic slippage of water inside single-wall nanotubes. The friction coefficient λ can be directly computed from equilibrium MD simulations using a well-known Green–Kubo relationship.^30^ In Figure 1 we show the dependence of λ on the tube diameter computed in this work for 16 different nanotubes as well as graphene and h-BN surfaces. Also shown is a—by no means comprehensive—selection of results obtained in previous work to illustrate the widespread of results, which we will address in detail below.

Based on our simulations, we find that irrespective of the curvature, water exhibits a ≈4–5 times larger friction coefficient on BN surfaces compared to equivalent carbon systems, reaching a maximum value of ≈4.5 × 10^4^ N s m^–3^ and ≈17 × 10^4^ N s m^–3^ for monolayer graphene and hBN, respectively. These friction coefficients on the curvature-free interfaces agree well with previous computational studies.^20,23,24,31−34^ In fact, our benchmark simulations provide a reliable estimate of the absolute values which are highly scattered ranging from ≈1 to ≈10 × 10^4^ N s m^–3^ (experiments^35^ report a friction coefficient of ≈12 × 10^4^ N s m^–3^ on graphite) and ≈4 to ≈30 × 10^4^ N s m^–3^ for the distinct systems. This wide spread of results can be associated with differences in the chosen force field,^31,33,36^ DFT functional,^23,24^ or simulation setup related to a frozen substrate,^20^ finite size errors, and thermostatting^37^ as well as confinement of water between two layers.^24,32^

In nanotubes, for both materials, a stark radius dependence is observed where smaller diameters lead to a significantly reduced friction of ≈1 × 10^4^ N s m^–3^ and ≈6 × 10^4^ N s m^–3^ for the smallest CNT and BNNT (radius ≈0.8 nm), respectively. As an illustration of the dimension of this effect, we highlight that the friction inside the smallest BNNT approaches the value of graphene which is generally considered to exhibit a large hydrodynamic slippage. For larger tube diameters, the friction coefficient converges to the value of the flat surface for both materials at radii ≳ 2.5 nm. This first-principle estimate is 1 order of magnitude smaller than observed in experiments^3^ and rather similar to findings of previous force-field-based simulations.^20^ In fact, the friction in CNTs predicted by our simulations generally exceeds the values obtained in nanofluidic measurements in isolated^3^ and membranes of multiwall^1,2^ CNTs. For BNNTs, moreover, we observe slippage of considerable extent opposed to the experiments.^3^ We will discuss these deviations between experiments and simulations in detail in a later section. For now, however, we focus on understanding the physical mechanisms behind the radius and material dependence observed in our reference simulations.

### Unveiling the Distinct Roles of Oxygens and Hydrogens in Water Transport

Solid–liquid friction is strongly determined by the (free) energy barriers that molecules have to overcome to move across the surface. Thus, we begin by examining the free energy surface (FES) of the water molecules in the contact layer to further understand the radius and material-dependent slippage. In particular, we investigate the overall corrugation of the FES with its square being proportional to the friction coefficient,^8,38^ such that λ ∝ (Δ*F*)^2^ (the exact relation is stated in the Supporting Information in section S1.C.2). In previous work,^20,23,24,39,40^ the analysis of the potential and free energy profiles has been limited to the oxygen atoms of the water molecules. With a recent study^33^ suggesting that the material dependence could be attributed to hydrogen–nitrogen interactions, here we examine the free energy barriers for both the oxygens and the hydrogens separately.

**Figure 2:**
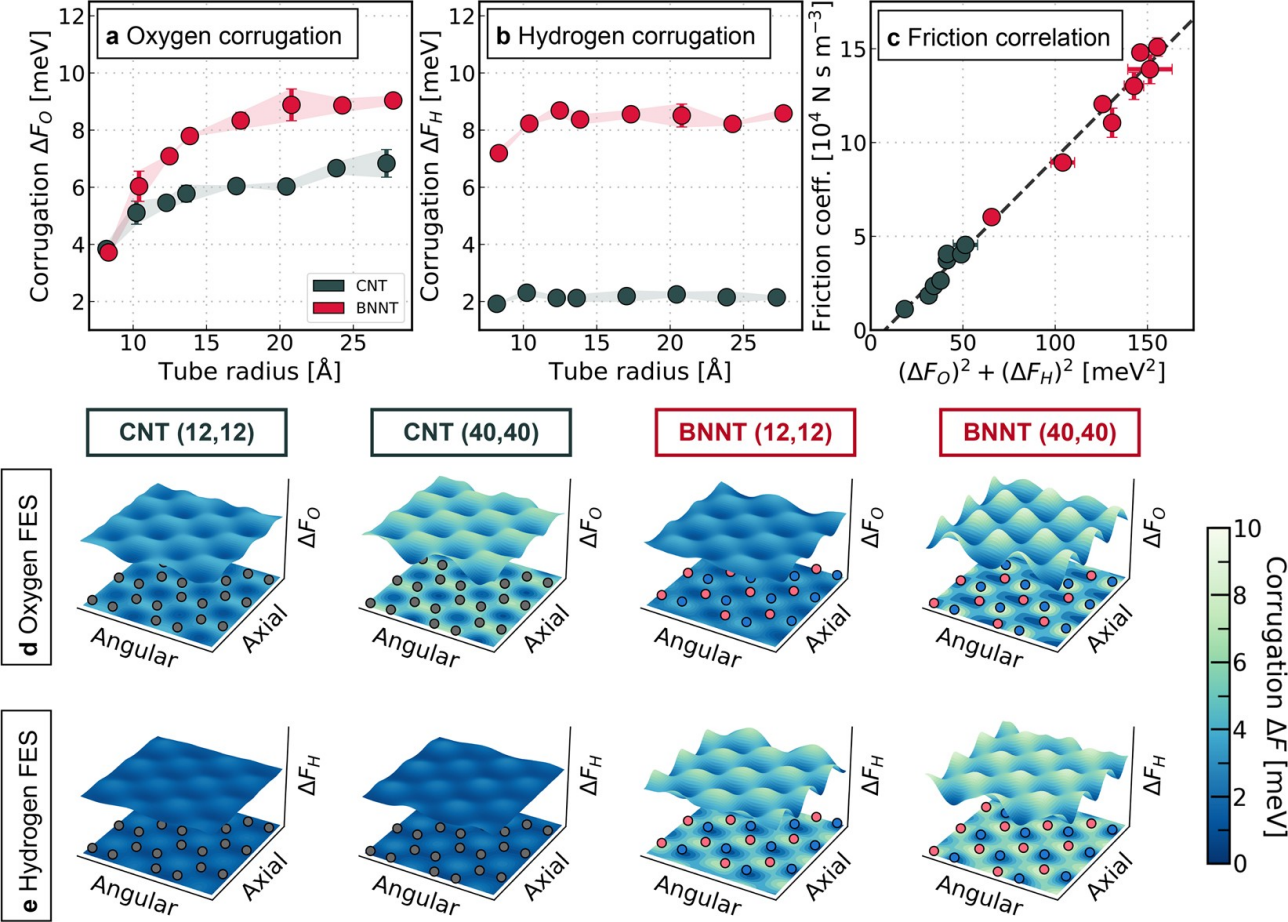
Linking the friction to the FES of water confined to CNTs and BNNTs. (a) Corrugation Δ*F*_O_ of the oxygen-based FES for CNTs and BNNTs plotted as a function of the tube radius. The error bars correspond to the statistical error that was obtained by splitting each trajectory into two blocks. (b) Corrugation Δ*F*_H_ of the hydrogen-based FES for CNTs and BNNTs plotted as a function of the tube radius. (c) Correlation between the friction coefficient and the sum of the squared corrugations. The dashed line represents a linear fit to the data obtained via orthogonal distance regression. (d) Visualization of the oxygen-based FES for the smallest and largest CNTs and BNNTs. The solid atoms are represented by the markers in the projection where carbon, boron, and nitrogen are colored in gray, pink, and blue. (e) Visualization of the hydrogen-based FES for the smallest and largest CNTs and BNNTs.

In Figure 2 we show how the FES of hydrogens and oxygens varies between materials and with curvature. To this end, we illustrate selected FESs for the smallest and largest CNTs and BNNTs investigated and plot the corrugation as a function of the tube diameter. Focusing on the oxygen corrugation (Figure 2a) and profiles (Figure 2d) at first, it is clear that the FES becomes more corrugated with increasing radius. The energetically favorable positions of the oxygens in the contact layer, conversely, do not vary with curvature and coincide with those observed on flat surfaces.^23^ On carbon surfaces, oxygen atoms preferentially sit on the hollow site in the middle of a hexagon of carbon atoms. At the BN interface, in addition to the hollow site, oxygen atoms show an additional free energy minimum around the boron atom. The minima observed agree with previous DFT^41^ and diffusion Monte Carlo (DMC) calculations for the flat graphene and h-BN sheets.^42,43^

Although the smoothening of the oxygen FES can qualitatively explain the radius dependence inside single-wall nanotubes, showing an almost identical corrugation for the smallest CNT and BNNT, it cannot justify a 5 times larger friction. In stark contrast to the oxygen atoms, the hydrogen-based FES features only a very weak radius dependence for both materials as shown in Figure 2b,e. Moreover, there is a pronounced difference between carbon and BN interfaces with the latter showing a roughly 4 times higher corrugation for the hydrogen FES. Interestingly, for the smallest BNNT, the corrugation of the hydrogen-FES is almost twice as large as that of the oxygen FES. Unsurprisingly, the hydrogen atoms preferably adopt the positions not occupied by the oxygens. In CNTs, this refers to the positions around the carbon atoms, while the free energy minimum is around the nitrogen atoms in BNNTs. The interaction between hydrogens and nitrogens in BNNTs is too weak to be classified as hydrogen bonding. However, it still yields an enhanced barrier for the water molecule to overcome, which explains the difference in friction observed for the smallest nanotubes. Here, we are able to quantify the magnitude of this effect by comparing it to the nonpolar carbon surfaces and show that it is almost independent of the curvature of the confinement. Further, we show in Figure 2c that there is a linear relation between the friction coefficient and the sum of the squared corrugation, highlighting the importance of accounting for the contributions from both oxygen and hydrogen.

**Figure 3:**
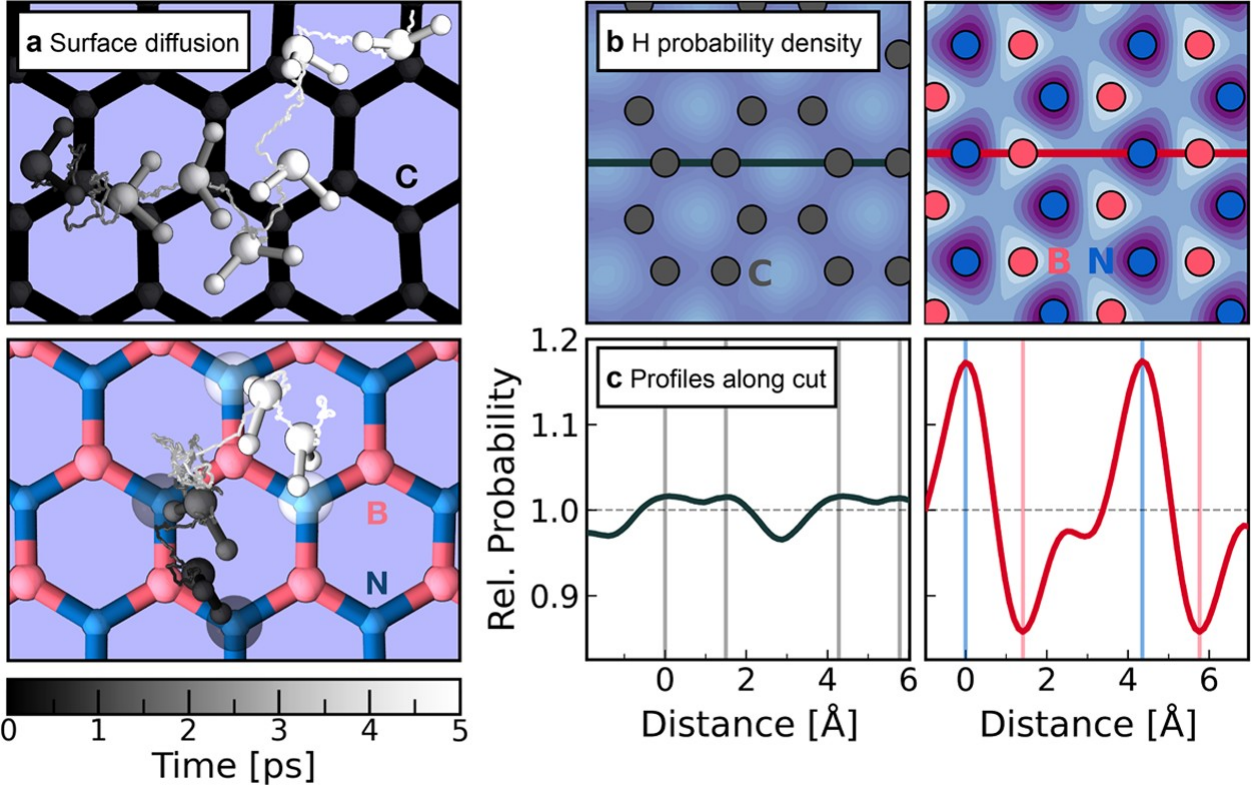
Transport mechanisms of water across carbon and BN surfaces. (a) Snapshots of the trajectory of an individual water molecule in the contact layer diffusing across graphene (top) and hBN (bottom). Both the path and individual snapshots of the water molecule are color-coded according to the time spanning overall 5 ps. In the bottom panel, the respective nitrogens involved in the docking events are colored according to the color of the water molecule at the given time. (b) Two-dimensional probability density of the hydrogen atoms in the contact layer on graphene (left) and hBN (right). For both materials, we use the identical scale of the color-coding where low and high probabilities correspond to light blue and dark purple, respectively. The colored markers represent the average position of the solid atoms, and the lines illustrate where the probability density is cut along for further analysis. (c) Profiles of the two-dimensional probability density along the cut directions for graphene (left) and hBN (right). The probability is expressed relative to the average probability of the respective system. The vertical lines represent the average position of the solid atoms shown in the panel above.

The results of our separate analysis of the FES point toward a distinct motion pattern on both surfaces, explaining the significantly larger friction in BNNTs compared to CNTs. To understand this mechanism, we follow the trajectory of individual water molecules in the contact layer next to the solid surface. It is worth noting, however, that while friction is a collective property, the surface diffusion is here investigated for individual atoms not accounting for mechanisms based on the collective motion of water molecules, as proposed in ref (44). Figure 3a illustrates this on the flat graphene and hBN sheet for a selected time period of 5 ps. As illustrated by the FES, the transport of water on graphene is mainly determined by the position of the oxygen with the orientation of the molecule being relatively unimportant. This rather unconstrained motion enables fast transport. On a hBN surface, conversely, the hydrogens play an important role and govern the diffusion path of the water molecule, as highlighted by the corrugation of the FES. Tracing individual water molecules, we observe a docking mechanism with water adopting a specific configuration (a so-called one leg structure)^41^ which fluctuates closely around the nearest nitrogen atom. The transport across the surface is then characterized by hopping events, where the water molecules perform jumps between nitrogen sites where they then have a longer residence time.

To provide further evidence of the identified hopping–docking diffusion scheme on BN surfaces, we show the two-dimensional probability density of the hydrogen atoms in the contact layer for graphene and hBN in Figure 3b. In contrast to an almost homogeneous distribution on graphene (left panel), the hydrogens preferably arrange above the nitrogen atoms on hBN. We now compare the profiles of the relative probability along a cut through the density as shown in Figure 3c. With this profile being linked to the transport mechanism, we indeed find a strongly corrugated pattern for hBN corresponding to an impeded reorientation. These findings also agree well with previous work^45−47^ where it was shown that water hydrogens approach nitrogen atoms in hBN considerably closer than the boron atoms or carbon atoms on graphene. This underlines the observed trends in the trajectories and indicates that this hydrogen–nitrogen interaction is indeed the culprit behind the material dependence. While the oxygen-driven diffusion of water molecules in CNTs enables a large hydrodynamic slippage, water transport in BNNTs is hydrogen dominated, resulting in a higher friction.

### Bridging the Gap between Simulations and Experiments

Having provided a clear and consistent picture for water transport in single-wall CNTs and BNNTs, we now return to discuss the evident deviations between our simulations and the nanofluidic measurements^1−3^ (Figure 1). While the chosen reference DFT functional as well as the water density inside the nanotubes can have an impact on the absolute values of the friction coefficient (see Supporting Information section S2.A and S2.D), it seems improbable that the observed discrepancies are exclusively caused by these parameters. Rather, the differences could, in principle, stem from effects not taken into consideration in both our simulations and the experiments. Starting with the discrepancies found for CNTs, one particularly interesting issue is the potential importance of a non-Born–Oppenheimer-based quantum friction that may play an important role in multiwalled CNTs.^44^ Specifically, it was suggested that this additional friction is induced by coupling of charge fluctuations in the water to the electronic excitations in the solid. With the electrons being able to tunnel between stacked layers, this additional term dominates water transport on graphite and multiwalled CNTs of large diameter where individual layers interact strongly.^48^ At smaller diameters, conversely, the weakening of the interlayer coupling results in a decreasing contribution of this quantum friction which then becomes negligible in single-wall CNTs and graphene. If quantum friction plays a significant role in multiwall nanotubes, then differences between our simulations on single-wall nanotubes and experimental measurements on multiwall nanotubes are to be expected. A second factor worth taking into consideration is the rigidity of the nanotubes and how this changes with radius and/or upon going from single-wall to multiwall nanotubes. Our simulations reveal a significant difference in the friction between frozen and flexible CNTs (see Supporting Information section S2.G and ref (37)). If the tube’s rigidity increases due to the enhanced interlayer coupling at larger diameter, then this could also significantly alter the friction, thus providing a classical explanation for the radius dependence in multiwall nanotubes. Going forward, it would therefore be interesting to explore multiwalled nanotubes with the ML framework exploited here as well as attempting to account for the non-Born–Oppenheimer electronic friction. In addition, experimental measurements for single-wall nanotubes and graphene would be particularly welcome.

BNNTs are considered next, which are considerably less slippy than CNTs. Experiments^3^ report a slip length of <5 nm for all BNNTs, providing a lower limit to the friction coefficient of ≈20 N s m^–3^. This agrees well with our findings for the large nanotubes and remaining discrepancies could stem from the high surface charge inside BNNTs observed in experiments.^16,49^ Recent computational studies based on DFT^50^ and AIMD simulations^51^ attribute this to the ability of hydroxide ions to bind to boron atoms. In highly alkaline water (high pH), the large number of chemisorbed ions on the surface could then impede the fluid transport by strongly interacting with the water molecules. Although we did not observe any dissociation of water molecules in our extensive simulations, the surface charge could be enhanced by defects in the confining material promoting dissociation and, thus, increasing the friction.^52^ While further investigations on the impact of pH and defects on the friction are required to determine the origin of the lack of flow in BNNTs, our simulations represent an important reference for the pristine surfaces indicating no sign of dissociation. These findings put stress on the experiments^3^ and underline the importance of extending the set of nanofluidic measurements in nanotubes.

## Conclusion

In conclusion, we have reported an extensive set of results from first-principles-based MLPs on the material and radius-dependent friction of water in single-wall nanotubes. To obtain a reliable description of water transport on low-dimensional materials, we developed a set of MLPs enabling us to simulate large-diameter nanotubes at first-principles accuracy. We find that the hydrodynamic slippage strongly depends on curvature for both materials and that there is a ≈5 times lower friction coefficient on carbon compared to BN. While differences from experiments remain, it is important to note that our benchmark data are based on pristine single-wall nanotubes, while the nanofluidic measurements were conducted in multiwalled and potentially defective nanotubes. By giving reliable values for water transport in defect-free single-wall nanotubes, our work represents a solid foundation to thoroughly understand hydrodynamic slippage while highlighting the lack of and need for additional experiments.

Beyond providing well-defined reference data, by achieving quantum mechanical accuracy, our simulations provide detailed insight into the origin behind the radius and material dependence of the water transport. To this end, we computed the free energy profile—separately for oxygen and hydrogen atoms—and find that the radius dependence of the friction is accompanied by a smoothing of the oxygen-based FES with decreasing tube diameter, reducing the energy barriers impeding fast transport. The sticky behavior of water on BN surfaces, conversely, can be traced back to their distinct chemistry and polarity impacting mostly the hydrogen atoms: While hydrogens experience low-energy barriers when water diffuses across a carbon surface, the hydrogen-based FES on BN surfaces is more corrugated and heterogeneous. Governed by the hydrogen–nitrogen interaction, the water molecules adapt an alternating hopping–docking motion inside BNNTs, translating into a larger friction compared to CNTs. By linking the transport behavior of water to this mechanism at the nanoscale, our work highlights the importance of the electronic structure of the substrate and provides an explanation of the radius and material dependence in pristine single-wall nanotubes. This clear knowledge of the mechanism behind the materials and radius dependence of water flow in nanotubes is expected to enable the design of tailor-made nanofluidic devices for directional flow or blue energy harvesting.

## Methodology

### Machine Learning Potentials

In this work, we build on the pioneering work of Behler and Parrinello^28,53^ and follow our recently introduced ML framework^29^ to develop and carefully validate committee neural network potentials (C-NNPs)^54^ for the water-carbon and water-BN systems, respectively. C-NNPs enable more accurate predictions than an individual NNP and, most importantly, grant access to an estimate of the error of the model provided by the disagreement between committee members. We train our potentials to energies and atomic forces obtained from DFT calculations within the generalized gradient approximation using the dispersion-corrected functional revPBE-D3.^55−57^ It is important to note that this level of theory has been shown to accurately reproduce both the experimentally measured structure and dynamics of liquid water^58−60^ as well as the interaction energies of water on graphene and inside CNTs obtained using more advanced methods such as DMC and coupled cluster theory.^61^ To ensure the applicability of our MLPs for all radii investigated, the configurations included in the training set range from bulk water and interfaces with zero curvature to highly confined water in nanotubes. All models have been trained using the open-source package N2P2.^62^

### Molecular Dynamics Simulations

All MD simulations were performed using the CP2K^63^ simulation package at a temperature of 300 K in the NVT ensemble. The temperature was kept constant using stochastic velocity rescaling thermostats,^64^ with separate thermostats for the solid and the liquid. To account for the coupling between the phonon modes of the confining material and the water vibrations,^65,66^ all atoms were treated as flexible. Dependent on the material and curvature, the system size varied between ≈960 and ≈8300 atoms. The number of water molecules inside the nanotubes was chosen so that the density was 1.0 g/cm^3^, corresponding to that of bulk water. For the graphene and hBN sheets, the water film height was roughly 35 Å. The simulation length varied with the number of atoms; however, for all systems investigated, a minimum sampling time of 1 ns was achieved. In total, more than 40 ns of first-principles ML data has been obtained for 18 systems (16 nanotubes). In addition, by performing an extensive set of rigorous tests and convergence checks, we ensure that our results and the main conclusions are robust with respect to system size effects and the length of the dynamical trajectories. Furthermore, by investigating the impact of the chosen DFT functional, water density, and nuclear quantum effects, we find that while absolute numbers might change, the relative trends observed are sustained. See the Supporting Information for details of these tests.

## Supporting Information Available

Video S1: different motion patterns of water molecules on graphene, tracing the individual water molecule shown in Figure 3 (MPG)
Video S2: different motion patterns of water molecules on hBN, tracing the individual water molecule shown in Figure 3 (MPG)
Section S1 provides a comprehensive overview of the methodology and computational details: the system setup and settings used in the MD simulations (S1.A), the development and validation of the MLPs (S1.B), as well as the computation of properties such as the friction coefficient and the FES (S1.C). Section S2 discusses how the friction coefficient is affected by certain aspects of the simulation and model. This involves the impact of the water density (S2.A), the system size (S2.B), the simulation time (S2.C), the hydrogen mass (S2.D), the chosen DFT functional (S2.E), nuclear quantum effects (S2.F), and the flexibility of the confining material (S2.G) (PDF)

## Supplementary Material

nn2c02784_si_001.mpg

nn2c02784_si_002.mpg

## Notes

A preprint version of this manuscript^67^ has been previously submitted to the arXiv preprint server accessible at https://arxiv.org/abs/2202.04955.
