# Supplementary material for: Water Flow in Single-Wall Nanotubes: Oxygen Makes It Slip, Hydrogen Makes It Stick
Source: ACS Nano. 2022 Jun 21;16(7):10775–82. doi: 10.1021/acsnano.2c02784 (PMC9331139; doi:10.1021/acsnano.2c02784)
Supplement: Supplementary file 3 — nn2c02784_si_003.pdf [file nn2c02784_si_003.pdf]

Supporting Information for:
Water flow in single-wall nanotubes: Oxygen makes it slip, hydrogen makes it stick

Fabian L. Thiemann[1,2,3], Christoph Schran[2,1], Patrick Rowe[2,1], Erich A. Müller[3], and Angelos Michaelides[2,1]

[1]Thomas Young Centre, London Centre for Nanotechnology, and Department of Physics and Astronomy, University College London, Gower Street, London, WC1E 6BT, United Kingdom
[2]Yusuf Hamied Department of Chemistry, University of Cambridge, Lensfield Road, Cambridge, CB2 1EW, United Kingdom
[3]Department of Chemical Engineering, Imperial College London, South Kensington Campus, London SW7 2AZ, United Kingdom

In this supplementary information, we provide additional details on certain aspects of the study reported in the manuscript. This includes a detailed summary of the computational details and methods as well as an analysis of the sensitivity of the results.

S1 Computational details **S2**
S1.A Molecular dynamics simulation S2
S1.A.1 System setup S2
S1.A.2 Simulation setup S4
S1.B Development of machine learning potentials S4
S1.B.1 Automated development of the MLP S5
S1.B.2 Validation of the MLP S6
S1.C Computation of properties S7
S1.C.1 Friction coefficient S7
S1.C.2 Free energy surface S9

S2 Sensitivity of the friction coefficient **S10**
S2.A Water density S10
S2.B System size S12
S2.C Simulation time S12
S2.D Hydrogen mass S13
S2.E DFT functional S13
S2.F Nuclear quantum effects S14
S2.G Tube flexibility S15

S1 Computational details

S1.A Molecular dynamics simulation

S1.A.1 System setup

All systems considered in this work were simulated in orthorombic simulation cells employing periodic boundary conditions in the axial direction (z) for nanotubes and along the in-plane directions (x, y) for the sheets of graphene and hBN. We studied diameters ranging from $\approx$ 16 Å to $\approx$ 55 Å corresponding to system sizes between $\approx$ 900 and $\approx$ 8300 atoms. Irrespective of the diameter, we used 12 axial repetitions for each nanotube yielding a consistent length of $\approx$ 30 Å in axial direction. The sheets, conversely, were represented by a 6×10 supercell with a length of the in-plane dimensions of $\approx$ 25 Å. All nanotubes were of armchair chirality (m,m) where m takes values between 12 and 40. Previous force-field-based simulations [1] have shown that water experiences the lowest friction inside carbon nanotubes of this chirality compared to zigzag (m,0) and chiral (m, $n \neq m$) systems. This upper boundary for the hydrodynamic slippage makes armchair nanotubes the ideal reference systems to investigate the material and radius dependence of the water friction. The water density inside the nanotubes was set to the bulk limit of 1.0 g/cm^3. On the graphene and hBN sheet, conversely, the height of the liquid water film was $\approx$ 35 Å being almost twice as large compared to a previous *ab initio* molecular dynamics (AIMD) study [2] corresponding to a total of $\approx$ 2500 atoms. For the sake of clarity, we provide an overview of the systems investigated in the manuscript in table S1 containing information about the number of atoms, number of water molecules, and simulation time.

**Topology**	**Simulation details**		**Illustration**
	Carbon surface	BN surface	
Nanotube (12,12)	R_{tube} = 0.82 nm<br>N_{atoms} = 966<br>N_{water} = 130<br>t_{sim} = 5 ns	R_{tube} = 0.83 nm<br>N_{atoms} = 984<br>N_{water} = 136<br>t_{sim} = 5 ns	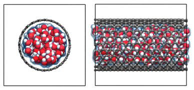
Nanotube (15,15)	R_{tube} = 1.02 nm<br>N_{atoms} = 1395<br>N_{water} = 225<br>t_{sim} = 2 ns	R_{tube} = 1.04 nm<br>N_{atoms} = 1434<br>N_{water} = 238<br>t_{sim} = 2 ns	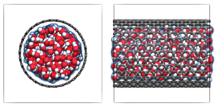
Nanotube (18,18)	R_{tube} = 1.23 nm<br>N_{atoms} = 1905<br>N_{water} = 347<br>t_{sim} = 2 ns	R_{tube} = 1.25 nm<br>N_{atoms} = 1962<br>N_{water} = 366<br>t_{sim} = 2 ns	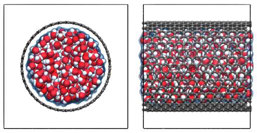

Nanotube (20,20)	$R_{tube} = 1.36$ nm<br>$N_{atoms} = 2286$<br>$N_{water} = 442$<br>$t_{sim} = 2$ ns	$R_{tube} = 1.38$ nm<br>$N_{atoms} = 2361$<br>$N_{water} = 467$<br>$t_{sim} = 2.25$ ns	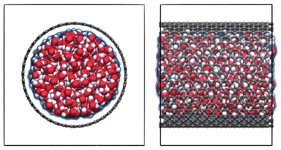
Nanotube (25,25)	$R_{tube} = 1.70$ nm<br>$N_{atoms} = 3392$<br>$N_{water} = 731$<br>$t_{sim} = 2$ ns	$R_{tube} = 1.73$ nm<br>$N_{atoms} = 3513$<br>$N_{water} = 771$<br>$t_{sim} = 2$ ns	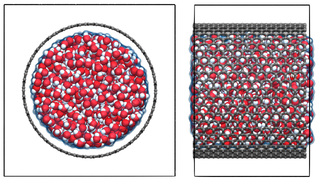
Nanotube (30,30)	$R_{tube} = 2.04$ nm<br>$N_{atoms} = 4716$<br>$N_{water} = 1092$<br>$t_{sim} = 1.4$ ns	$R_{tube} = 2.08$ nm<br>$N_{atoms} = 4884$<br>$N_{water} = 1148$<br>$t_{sim} = 1.2$ ns	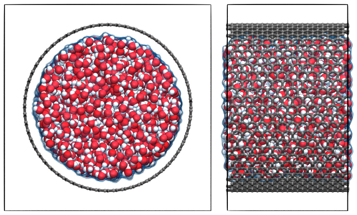
Nanotube (35,35)	$R_{tube} = 2.38$ nm<br>$N_{atoms} = 6255$<br>$N_{water} = 1525$<br>$t_{sim} = 2$ ns	$R_{tube} = 2.42$ nm<br>$N_{atoms} = 6501$<br>$N_{water} = 1607$<br>$t_{sim} = 1$ ns	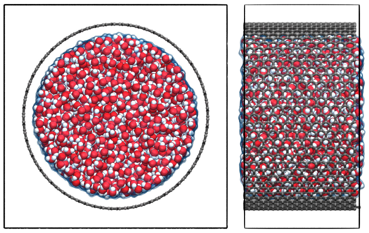
Nanotube (40,40)	$R_{tube} = 2.72$ nm<br>$N_{atoms} = 8004$<br>$N_{water} = 2028$<br>$t_{sim} = 1.3$ ns	$R_{tube} = 2.77$ nm<br>$N_{atoms} = 8328$<br>$N_{water} = 2136$<br>$t_{sim} = 1$ ns	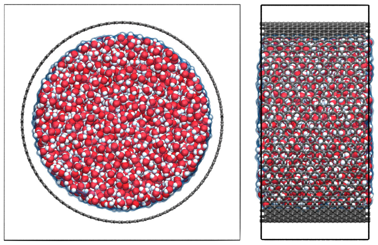
Sheet	$H_{film} = 3.5$ nm<br>$N_{atoms} = 2460$<br>$N_{water} = 740$<br>$t_{sim} = 2.25$ ns	$H_{film} = 3.5$ nm<br>$N_{atoms} = 2532$<br>$N_{water} = 764$<br>$t_{sim} = 2$ ns	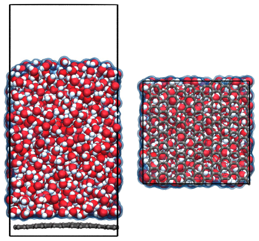

Table S1: Detailed overview of systems investigated in this work. For each system, we report the total number of atoms, N_{atoms}, the number of water molecules, N_{water}, and the simulation length, t_{sim}. For nanotubes and the flat sheet we further list the tube radii, R_{tube}, and water film height, H_{film}, respectively. In the right column, we show a front and side (bottom for the sheet) view of each system. The black lines represent the edges of the simulation box.

S1.A.2 Simulation setup

For all molecular dynamics (MD) simulations, we employed the CP2K [3] simulation package. In this work, we performed three different types of MD simulations: (i) *ab initio* MD (AIMD) to generate training data for the development of our machine learning potentials (MLPs); (ii) MD simulations using classical nuclei employing MLPs to generate the results reported in the manuscript; (iii) Path integral MD (PIMD) simulations with quantum nuclei to investigate the impact of nuclear quantum effects for the friction coefficient. In all three types of simulations, all atoms were treated as flexible and the center of mass velocity as well as the total angular momentum velocity were zeroed. Moreover, deuterium masses were employed for the hydrogens to ensure a stable simulations at computationally feasible timesteps. Convergence checks of a previous AIMD study of the water transport on graphene [2] have shown that the values obtained for the friction for H_2O and D_2O did not differ significantly and are within each other's error bars.

AIMD simulations

The AIMD simulations of CNTs and BNNTs filled with water densities 0.6, 0.8, and 1.0 g/cm^3 were performed using Langevin MD at 330 K employing using a timestep of 1 fs. We used the dual-space Goedecker-Tetter-Hutter pseudopotentials [4] to represent the atomic cores and the Kohn-Sham orbitals were expanded in DVZP basis set of shorter range (SR) [5]. In the original AIMD simulations a cutoff of 460 Ry was applied and the generalised gradient approximation (GGA) functional PBE [6] with the D3 dispersion correction [7, 8] was used. Within the training process of the MLPs, additional single point calculations were performed for the selected configurations where the cutoff was increased to 1050 Ry and the revPBE functional [9] with dispersion correction D3 was employed. The length of all AIMD simulations was above 100 ps.

MD simulations with classical nuclei

Irrespective of the material and curvature, all simulations were performed in the NVT ensemble where the temperature was set to 300 K. We employed two stochastic velocity rescaling thermostats [10] where one thermostat was applied to the liquid and solid, respectively. The timestep was set to 1 fs. To ensure that dynamical properties were not affected by the thermostats, the time constant was set to 1 ps for both thermostats. Each system was equilibrated for 50 ps before statistics were sampled for at least 1 ns.

PIMD simulations with quantum nuclei

Path integral MD simulations in the NVT ensemble at 300 K were performed via thermostat ring polymer MD (TRPMD) [11] employing the PILE thermostat [12]. Using deuterium instead of hydrogen allows us to achieve converged properties with 16 beads while employing a timestep of 0.25 fs. Due to the increased cost, simulations were performed for a reduced simulation time of 500 ps and using 6 instead of 12 replicas of the unit cell in axial direction. To compute the friction coefficient, the summed force per frame was averaged over all beads which was then correlated to apply the established Green-Kubo relation.

S1.B Development of machine learning potentials

Our MLPs for water in CNTs and BNNTs rely on Behler-Parrinello neural network potentials (NNPs) [13, 14] in order to form a committee neural network potential (C-NNP) [15]. The NNP formalism has been successfully employed to understand the unique properties of water [16, 17] and was also used to study

water flow in hBN channels [18], making it the ideal method for the current study. Furthermore, we have recently shown that the C-NNP methodology enables the simple generation of MLPs for complex aqueous systems [19]. The main idea behind the C-NNP approach is the combination of multiple NNPs in a "committee model", where the committee members are separately trained from independent random initial conditions to a subset of the total training set. While the committee average provides more accurate predictions than the individual NNPs, the committee disagreement, defined as the standard deviation between the committee members, grants access to an estimate of the error of the model. This committee disagreement provides an objective measure of the accuracy of the underlying model. To construct a training set of such a model in an automated and data-driven way, new configurations with the highest disagreement can be added to the training set. This is an active learning strategy called query by committee (QbC) and can be used to systematically improve a machine learning model. For further details on the C-NNP methodology we refer the reader to Ref. 15.

S1.B.1 Automated development of the MLP

For the development of two MLPs we used our established active learning workflow [15, 19], which is split into different generations. In each generation new state points are targeted to yield a C-NNP that will be used to generate new candidate structures for the next generation. This strategy relies on the fact that a C-NNP model can faithfully yield configurations even for thermodynamic conditions not yet considered in the training set, if the conditions are not drastically different from those already considered. Within a generation, QbC is used to adaptively extend the training set by selecting the most representative configurations separately for each state point, improving its description. With this procedure, multiple state points can easily be treated in parallel. At the very beginning of each QbC cycle, a small number of random configurations is chosen to train the first committee, while in subsequent iterations, new configurations are selected based on the highest atomic force committee disagreement. Convergence of these individual QbC cycles can be detected by monitoring this disagreement. Once all QbC cycles for the selected conditions in a given generation are converged, the individual training sets are combined and a final tight optimization of that generation's resulting NNP is performed.

Using this workflow as summarised in Fig. S1, we have first targeted small (12,12) carbon and hBN nanotubes filled with water densities of 0.6, 0.8 and 1.0 g/cm^3. The initial models were seeded by short AIMD simulations at 330 K, providing after active learning the first generation models with 465 and 387 structures for carbon-water and hBN-water, respectively. In the second generation, bulk water and water on flat graphene and hBN sheets were targeted by individual active learning cycles. The required structures were generated from C-NNP simulations at 330 K with a minimum length of 200 ps for both materials. Bulk water was simulated in a periodic box of 64 water molecules at experimental equilibrium density, while water in contact with the flat sheets was described by 130 and 136 water molecules on a 3×5 supercell of graphene and hBN, respectively. Overall, for both materials 148 new structures from these conditions were added to the training sets of the two models. To incorporate the quantum nature of the nuclei into the description of our C-NNP models, we performed PIMD simulations in the next generation. For that purpose, we performed the same simulations as before with quantum nuclei using the two C-NNP models, resulting in 5 distinct conditions which have been targeted by separate active learning cycles. During these cycles 483 and 350 additional structures were identified and added to the training set.

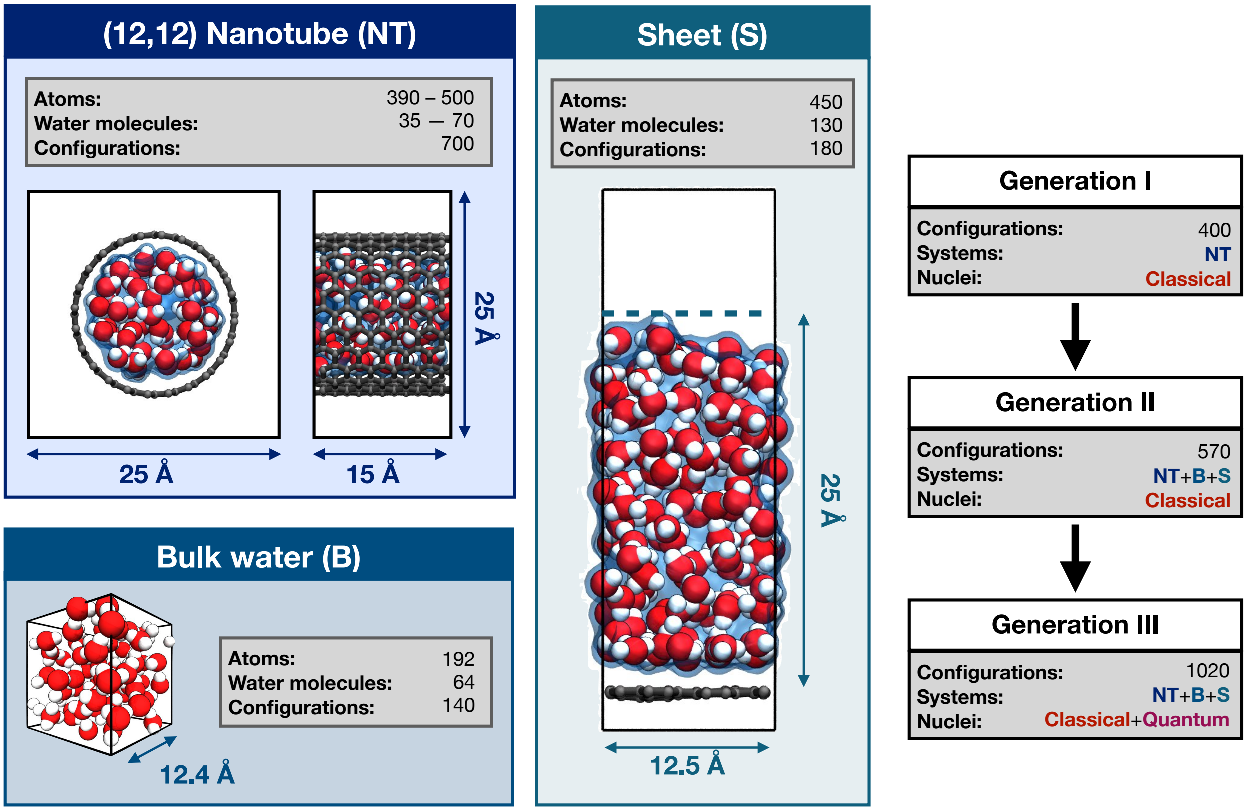

Figure S1: Overview of development of the MLP summarising the systems included in the stepwise active learning process. The left part shows the three different types of systems incorporated in the model. These include a nanotube (NT) with chirality (12,12) filled with water at densities $\rho \in [0.6, 0.8, 1.0]$ g/cm^3, bulk water (B), and a water film on top of a sheet (S). For each of these systems, we show snapshots highlighting the relevant dimensions and giving information about the total number of atoms, the number of water molecules, as well as how many configurations of each system were included in the training of the final models. On the right, we show how the model is improved in a stepwise manner with our active learning workflow. In Generation I, the model is exclusively build on configurations extracted from AIMD trajectories of water-filled nanotubes. Using this model as a computationally efficient structure generator, the training-set in generation II is extended by actively selecting individual structures from classical MD simulations performed with the model of generation I. In a final step, the model from generation II is employed to perform PIMD and configurations accounting for nuclear quantum effects (NQEs) are added to the training set of final model called generation III. For all generations, the configurations to be included in the training process where selected by QbC.

S1.B.2 Validation of the MLP

The final training sets consist of 1096 and 885 structures for CNNTs and BNNTs, respectively, while spanning diverse conditions and including both a classical and quantum description of the nuclei as shown in figure S1.

For our carbon-water C-NNP model we obtain an energy and force root mean square error (RMSE) for the training set of 0.8 meV per atom and 72 meV/Å, respectively. The hBN-water C-NNP model was trained with an energy and force RMSE value of 0.7 meV per atom and 47 meV/Å, respectively. To test the performance of the model for the study of water flow in single-wall nanotubes of different radii, we build an out-of-sample validation set by drawing configurations from our production simulations for the 12-12, 20-20 and 30-30 nanotubes as well as water on the flat graphene and hBN surfaces. Evaluating the DFT energies

and forces for 50 randomly selected structures of each system allows us to assess the accuracy of our models in application to faithfully validate the main findings of this study. The performance of our two models over this diverse set of validation structures is summarised in figure S2. The largest force RMSE for this validation set is 56.6 meV/Å, suggesting that the model remains accurate and robust across the wide range of conditions and systems studied here.

To further validate the performance of our model for the description of water, we ran bulk water simulations at ambient conditions and compare to published [20] structural and dynamical properties obtained with our reference DFT setup as shown in Fig.S3. To achieve a fair comparison, hydrogen masses were adopted instead of deuterium and the timestep was decreased to 0.5 fs. For both models, the simulation time was 1 ns. The radial distribution functions (RDF) obtained with both our carbon-water and hBN-water model are in near perfect agreement with the published results of the revPBE-D3 functional [7–9]. Furthermore, following the procedure outlined in reference 20 we computed the diffusion constants of water based on a linear fit to the mean square displacement of the molecular center of mass in the time interval between 1 and 10 ps. The correction introduced by Yeh and Hummer was applied to account for finite size effects 21. Using these settings, we find that both models are in very good agreement with the reference, accentuating the high accuracy of our model.

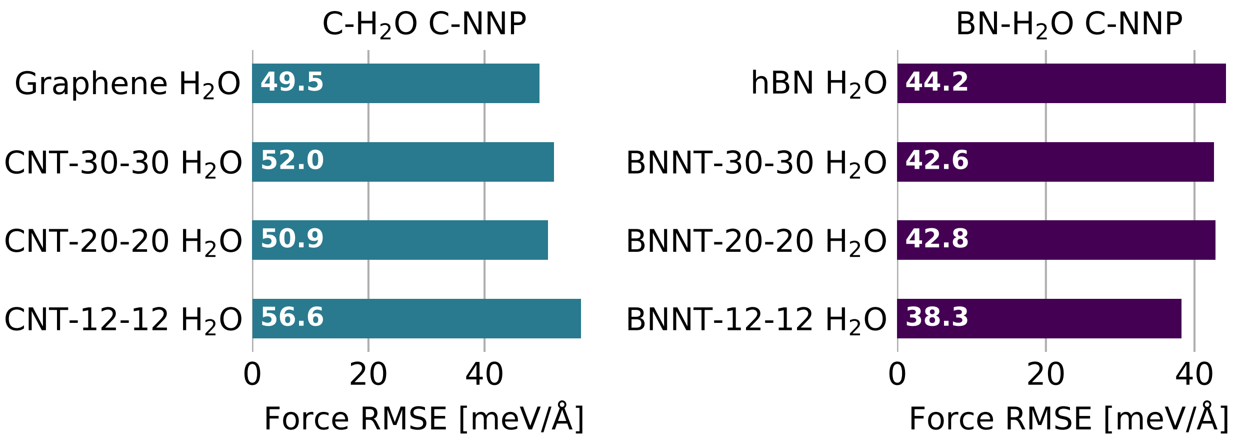

Figure S2: Performance of the carbon-water and hBN-water C-NNP models for the diverse validation sets. The left panel summarises the force accuracy of carbon-water C-NNP model, while the right panel features the accuracy of the hBN-water model. For each model a diverse validation set was constructed from our production simulations for nanotubes of various size as well as water in contact with the flat sheets. .

S1.C Computation of properties

S1.C.1 Friction coefficient

The friction coefficient λ is defined as proportionality constant between the total force of the solid exerted on the liquid parallel to the surface, $\mathbf{F}_{\mathrm{wall}}$, normalised by the surface area, A, and the fluid velocity at the interface, $\mathbf{v}_{\mathrm{wall}}$,

$$\lambda \mathbf{v}_{\mathrm{wall}} = \frac{\mathbf{F}_{\mathrm{wall}}}{A} \ . \tag{1}$$

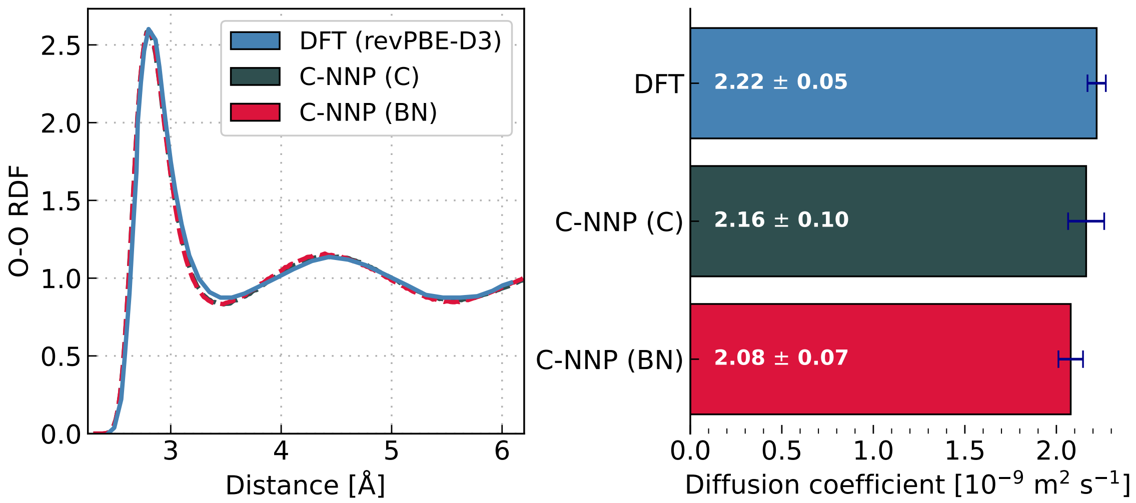

Figure S3: Properties of bulk water predicted by the published simulations [20] performed with the chosen DFT functional and by our C-NNPs. The left panel shows the oxygen radial distribution function (RDF) and on the right we illustrate the performance for the diffusion coefficient. The error bars correspond to the statistical error obtained from splitting the entire trajectory into two blocks. .

Based on linear response theory, the friction coefficient can be readily computed from equilibrium MD simulations using a Green-Kubo relation [22, 23]

$$\lambda_{\mathrm{GK}}(t) = \frac{1}{Ak_{\mathrm{B}}T}\int_0^t \langle \mathbf{F}_{\mathrm{wall}}(t') \cdot \mathbf{F}_{\mathrm{wall}}(0)\rangle dt' \ , \tag{2}$$

where k_{B} is the Boltzmann constant, T corresponds to the temperature, and the brackets correspond to an ensemble average. The total force of the solid, $\mathbf{F}_{\mathrm{wall}}$, given by the summed force of all solid atoms of a given configuration, is saved every timestep (1 fs). For nanotubes, only the force components in axial (z) direction were used to compute the friction. In the case of graphene and hBN, conversely, $\mathbf{F}_{\mathrm{wall}}$ was evaluated for both in-plane dimensions (x, y). The friction coefficient of the system is then recovered for sufficiently long time intervals, $\lambda = \lim_{t\to\infty} \lambda_{\mathrm{GK}}(t)$. In practice, however, rather than reaching a plateau at infinite time the Green-Kubo integrals, $\lambda_{\mathrm{GK}}(t)$, vanish for systems of finite size [24] complicating its accurate evaluation. In this work, we circumvent this so-called "plateau problem" [25] by following a recently introduced methodology [26] where an analytical expression

$$\Lambda(t) = \lambda_0 \left(e^{-\frac{t}{t_1}} - e^{-\frac{t}{t_2}}\right) \tag{3}$$

is fitted to the Green-Kubo integrals obtained from simulations. The finite-size-corrected friction coefficient, λ, can then be computed from the three fitting parameters, λ_0, t_1, and t_2 based on

$$\lambda = \lambda_0 \frac{1-u}{1+u} \ , \tag{4}$$

with $u = t_2/t_1$. For all systems, the Green-Kubo integrals were computed for a correlation time of 2.5 ps while an R^2 value above 0.85 for the fit of the analytical expression from reference [26] was ensured. We illustrate this procedure in figure S4 by showing the Green-Kubo integrals as well as the fitted functions for smallest CNT and BNNT.

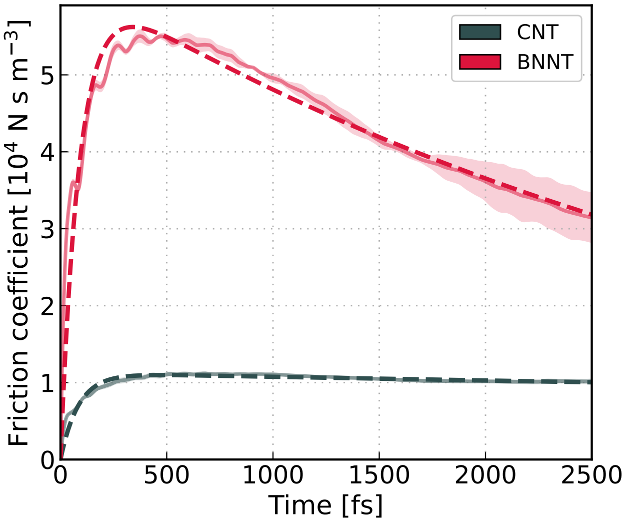

Figure S4: Circumventing the "plateau problem" for finite size systems in the Green-Kubo formalism. The continuous lines correspond to the Green-Kubo integrals, $\lambda_{\mathrm{GK}}(t)$, obtained from our MD simulations of the smallest CNT and BNNT. The dashed lines, conversely, represent a fit of equation 3 to the simulation data. The true friction coefficient can then be extracted based on the fitting parameters according to equation 4.

S1.C.2 Free energy surface

In the manuscript, we link the friction coefficient λ to the corrugation of the free energy surface (FES) ΔF of the oxygen and hydrogen atoms in the contact layer. Based on theoretical considerations [27, 28], the friction coefficient can be approximated in terms of the structure and dynamics of the liquid water contact layer such that

$$\lambda \approx \frac{N_l}{A k_{\mathrm{B}} T} (f_{\mathbf{q}_{\parallel}})^2 \cdot S(\mathbf{q}_{\parallel}) \cdot \tau_\rho \ , \tag{5}$$

where N_l corresponds to the water molecules in the contact layer, $\mathbf{q}_{\parallel}$ is the reciprocal lattice vector along the wall with a magnitude $|\mathbf{q}_{\parallel}| = 2\pi/a_0$ with a_0 being the lattice constant in real space, $f_{\mathbf{q}_{\parallel}}$ is the first Fourier component of the force between a water molecule and the substrate, $S(\mathbf{q}_{\parallel})$ is the static structure factor, and τ_ρ represents the density correlation time given by

$$\tau_\rho = \int_0^\infty \mathrm{d}t \frac{\langle \rho_{\mathbf{q}_{\parallel}(\mathbf{t})} \rho_{-\mathbf{q}_{\parallel}}(0) \rangle}{\langle \rho_{\mathbf{q}_{\parallel}}(0) \rho_{-\mathbf{q}_{\parallel}}(0) \rangle} \ , \tag{6}$$

with $\rho_{\mathbf{q}_{\parallel}}(t)$ being the Fourier component of the water density. Previous studies based on classical force fields [29] and AIMD [30] suggest that the dynamical contribution, namely the density decorrelation time τ_ρ, only weakly depends on the curvature and hardly differs between hBN and graphene. In this work, therefore, we focus on the static part, where $f_{\mathbf{q}_{\parallel}} = \frac{2\pi}{a_0} \Delta V$ is directly related to the corrugation of the potential energy surface ΔV which is typically approximated by ΔF culminating in the quadratic dependence, $\lambda \propto (\Delta F)^2$, mentioned in the manuscript.

Here, we provide a detailed explanation of the methodology behind the computation of the free energy profiles. Following previous work [2, 30], the two-dimensional FES is given by $\Delta F(x,y) = -k_B T \ln p_i(x,y)$, where k_B is the Boltzmann constant, T represents the temperature (here 300 K), and $p_i(x,y)$ corresponds to the normalised two-dimensional probability of finding species $i \in [\mathrm{O}, \mathrm{H}]$ in the contact layer at a point (x,y).

With nanotubes being of one-dimensional character, the coordinates x and y correspond to the azimutal and axial direction. Based on these definitions, computing the FES and related properties requires (i) a consistent definition of the contact layer, (ii) the efficient sampling of $p_i(x, y)$ in cylindrical-like systems, and (iii) an appropriate measure quantifying the corrugation itself.

Starting with the contact layer, we computed the distribution of the distance between the oxygen of each water molecule j and the closest solid atoms (C, B, N), $d^j_{\mathrm{O-X}}$. Interestingly, we found that for all systems the first minimum was located at a distance of ≈ 4.8Å. Therefore, we assign a water molecule j to the contact layer if $d^j_{\mathrm{O-X}} \leq 4.8$Å. Going through the configurations of a trajectory, the positions of either the oxygens or hydrogens - dependent on the species for which the FES is computed - of water molecules satisfying the criterion above are selected. It is important to note that for the hydrogen-based FES, both hydrogens of each water molecule in the contact layer are considered and contribute towards the density profile. By decomposing these coordinates into an axial and angular contribution, these positions are projected onto a two-dimensional grid corresponding to the nanotube opened up along a cut in axial direction. At this point, we already have an estimate of $p_i(x, y)$, however, we can accelerate its convergence by mapping $p_i(x, y)$ of the entire tube onto its unit cell. Making use of the inherent periodicity of the crystal lattice of the nanotube allows to extract 30-480 times more statistics per configuration dependening on the system. Following this procedure and by choosing a high sampling frequency of 20 fs, we obtain a converged estimate of $p_i(x, y)$ and, thus, the FES. At last, we are only left with defining the corrugation of the FES. In analogy to previous work [2, 30], this is done by taking the highest free energy present in the FES. However, to ensure this quantity is independent of noise we smooth the FES in advance by applying a two-dimensional Savitzky-Golay filter as implemented in common python libraries such as SciPy [31].

S2 Sensitivity of the friction coefficient

To ensure that the main results reported in the manuscript are invariant with respect to simulation settings, in this section we perform a sensitivity analysis of the friction coefficient. If not stated otherwise, the simulation time for each system is at least 1 ns.

S2.A Water density

To investigate the impact of the water density on the friction coefficient we performed additional simulations on the smallest nanotubes (12,12) varying the water density. A summary of this analysis is shown in the left panel of figure S5. For all nanotubes, the density, ρ, is computed as the ratio of water molecules, N, and the accessible volume, V, inside the tube. Specifically, V defines the volume of a cylinder of the length of the nanotube, l_{tube}, and the radius $\bar{r} = r_{\mathrm{tube}} - r_{\mathrm{VdW}}$ corresponding to the radius of the nanotube, r_{tube}, reduced by the Van der Waals radius, r_{VdW}, of the solid (≈ 1.7Å). We observe that for both materials the friction remains constant for a density $\rho \leq 0.8$ g/cm^3 resulting in values of $\approx 0.5 \cdot 10^4$ N s m^{-3} and $\approx 3.0 \cdot 10^4$ N s m^{-3} for the CNT and BNNT, respectively. At larger densities, conversely, the friction coefficient increases significantly reaching a five-fold enhancement at the highest density of 1.2 g/cm^3. Comparing the mass density profiles as a function of the radial distance illustrated in the right panel of figure S5 gives insight into the origin of the enhancement of the friction. In case of densities $\rho \leq 0.8$ g/cm^3, the water is in the liquid state. When the number of water molecules inside the tubes increases, however, the fluid becomes more structured (see the curves for $\rho = 0.9$ g/cm^3) and eventually freezes. This applies in particular to densities $\rho \geq 1.0$ g/cm^3

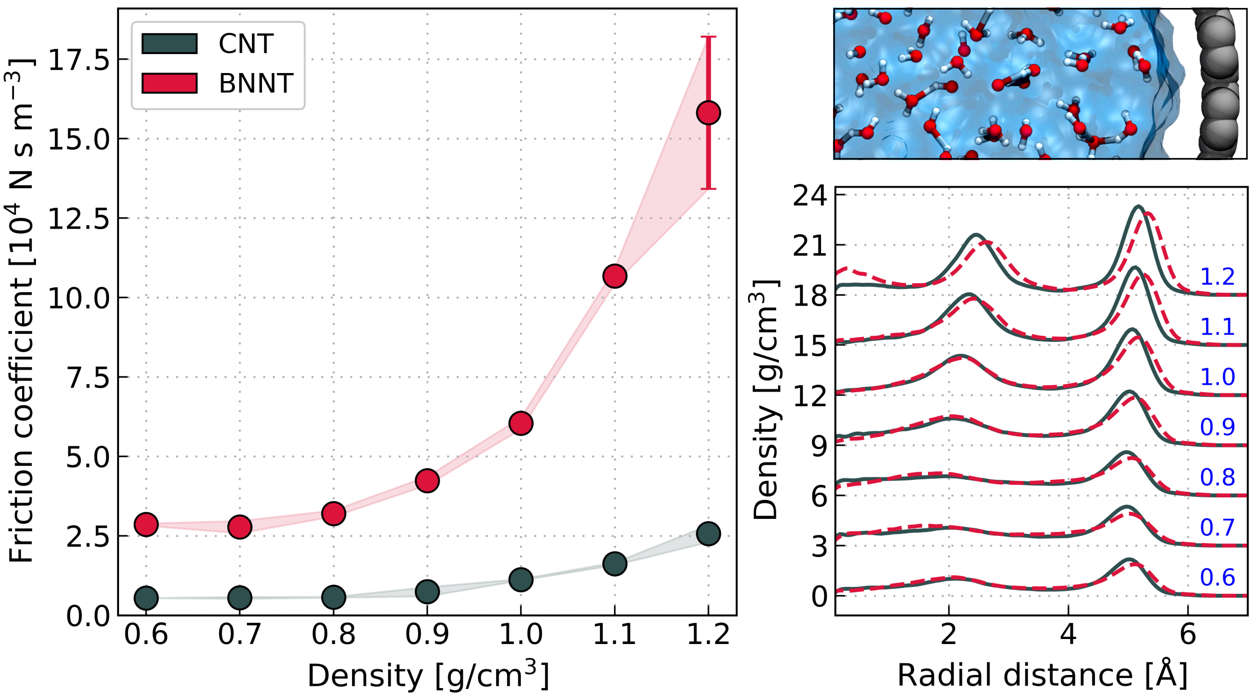

Figure S5: Impact of the water density on the friction coefficient. The left panel shows the friction coefficient computed for the smallest nanotubes (12,12) as function of the density. The error bars correspond to the statistical error obtained from splitting the entire trajectory into two blocks. In the right panel we show at the bottom the density profiles for the different densities analysed as a function of the radial distance from the tube center of mass. For the sake of clarity, the profile are shift by a factor of 3 along the y-axis with increasing density. The continuous lines correspond to CNTs while BNNTs are plotted using dashed lines. The top panel shows a snapshot of a CNT to illustrate the radial dependence more clearly.

where a second peak close to the tube center becomes apparent indicating the formation of a double-ring structure. The observation that a phase transition to the solid state induces a significantly larger friction is of general interest and could spark future research. With respect to this work, however, we lack knowledge of the physical water density inside the tubes of varying diameters at equilibrium. In principle, the accurate number of water molecules in each tube could be determined by performing either Grand canonical Monte Carlo (GCMC) simulations or connecting the nanotube to two reservoirs as done in previous work [29, 32]. Rather than performing these expensive and from the MLP perspective challenging simulations, here we employ the approximation of setting the density in all nanotubes to the bulk limit of 1.0 g/cm^3. While this approach might seem a bit crude, we note that (i) the difference between the materials hardly changes throughout all densities studied and (ii) the error associated with this approximation decreases with tube radius. The validity of this approach is supported, moreover, by the previous simulation studies based on classical force fields [29, 32] where the nanotubes were initially connected to water reservoirs to obtain the appropriate number of water molecules inside the tubes; the radial density profiles obtained therein show that after two structured water layers in vicinity to the wall the density indeed decays to the bulk limit for larger distances. Therefore, we expect that the main findings presented in the manuscript still hold despite the change of the absolute values.

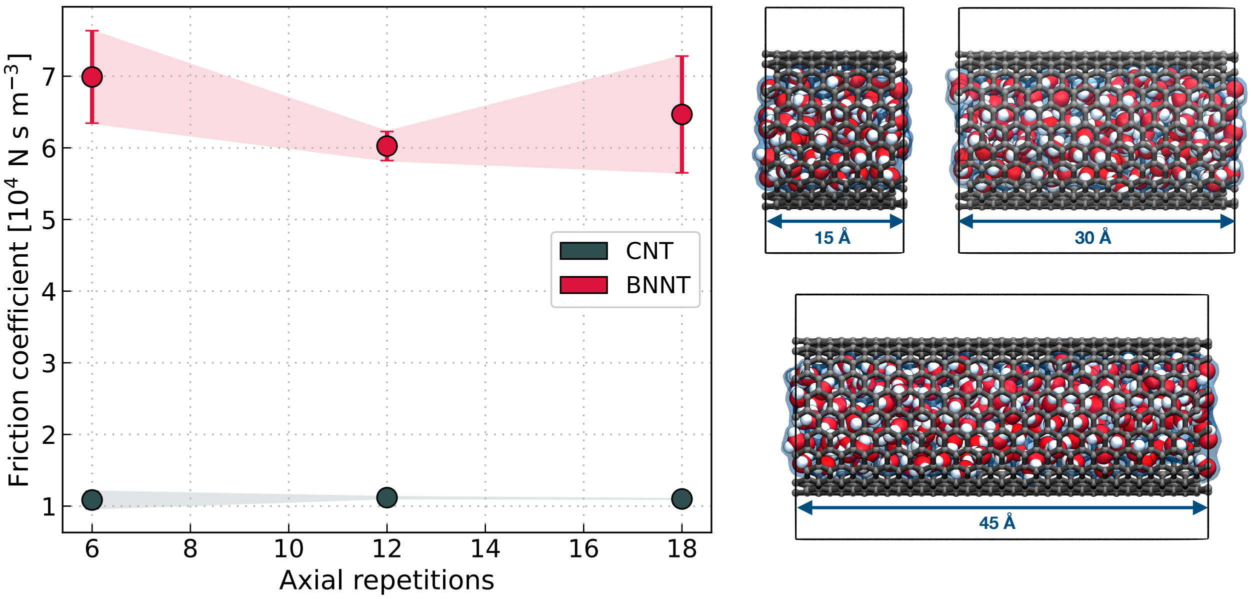

Figure S6: Dependence of the friction coefficient on the system size. The system size is quantified by the number of repetitions of the unit cell of a nanotube in axial direction corresponding to ≈ 2.5 Å. The three systems shown here, correspond to an axial length of ≈ 15, 30, and 45 Å comprising roughly 500, 1000, and 1500 atoms, respectively. The error bars correspond to the statistical error obtained from splitting the entire trajectory into two blocks. On the right, we show snapshots of the different tubes where arrows illustrate the axial length.

S2.B System size

While the usage of MLPs allows to reach significantly longer time and length scales, the simulation of the large diameter nanotubes are still computationally demanding. Therefore, for all nanotubes the box length in axial direction (z) is set to repetitions of the unit cell of the nanotube corresponding to ≈ 30 Å. In figure S6, we show based on the smallest nanotubes (12,12) that this size is indeed sufficient to obtain converged results for the friction coefficient. For both materials, the additionally simulations on cells comprising 6 and 18 axial repetitions, respectively, yield almost identical results for the friction of water inside the nanotubes. With results remaining unchanged even for an axial dimension smaller than the tube diameter, we are confident that the employed system size is sufficient to obtain converged friction coefficients even for the largest nanotubes.

S2.C Simulation time

In analogy to analysing the impact of the system size, here we explore the convergence of the friction coefficient with respect to the simulation length. To this end, we computed the friction coefficient for the smallest nanotubes (12,12) by varying the production time between 100 ps and 5 ns as shown in figure S7. Similar to the results presented in the previous section, the friction coefficient is found to change relatively little with increasing simulation time. For both materials, trajectories of the short length of 100 ps are already sufficient to converge the friction within ≈ 85% with respect to the value obtained from the reference simulation of 5 ns. While these short simulations have relatively large statistical errors, this convergence check shows that previous AIMD simulations of comparable simulation length [2, 30] can indeed provide reliable results within the given uncertainty. For our work, conversely, where all systems have been simulated

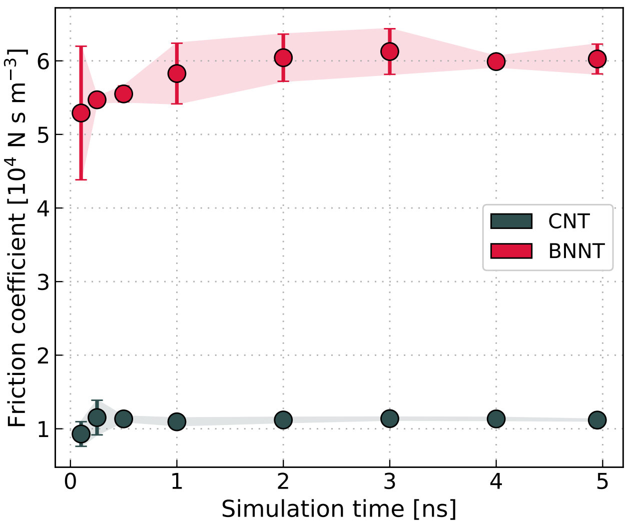

Figure S7: Convergence of the friction coefficient with simulation time. The error bars correspond to the statistical error obtained from splitting the trajectory of the given length into two blocks.

for at least 1 ns this analysis confirms that the estimates of the friction coefficients are reliable and converged with respect to the length of the trajectory.

S2.D Hydrogen mass

Here, we analyse the impact of employing deuterium masses for the hydrogen atoms on the friction coefficient. We performed additional simulations of the smallest nanotubes (12,12) using hydrogen masses instead of deuterium as used for all results reported in the main text. To enable stable simulations, we reduced the timestep in these simulations to 0.5 fs and subsequently computed the friction coefficient. A comparison of the obtained friction coefficients for D_2O and H_2O is shown in figure S8. In line with previous findings [2], our simulations indicate that the hydrodynamic slippage does not depend significantly on the hydrogen mass. However, it is interesting to note that the difference – albeit small – is more pronounced in case of the BNNT. In fact, this could be related to the observed hopping-docking mechanism which is dictated by the hydrogen-nitrogen interaction. Notwithstanding this observation, the presented findings underline the validity of employing D_2O instead of H_2O in our simulations enabling the application of a larger timestep.

S2.E DFT functional

In this subsection, we investigate how the friction coefficient of water changes with the chosen DFT functional. To this end, we took the training set of our MLP and recomputed energies and forces based on the hybrid functional PBE0 [33, 34] and the improved dispersion correction D4 [35–37]. It is important to note that, similar to its GGA analogue PBE [38], we found that PBE0-D4 predicts a significant overstructured liquid water in the bulk phase. This is distinctly different from the structure obtained with revPBE-D3 which is in close agreement with experiments [20]. Based on this functional and the associated new dataset, we trained new MLPs for the two materials investigated in this study. To analyse the dependence of the friction coefficient on the chosen level of theory, we then perform additional simulations on the smallest nanotubes (12,12), compute the friction coefficients, and compare them to those obtained with our models based on revPBE-D3. A summary of this comparison is shown in the left panel of figure S9. Interestingly, the hybrid

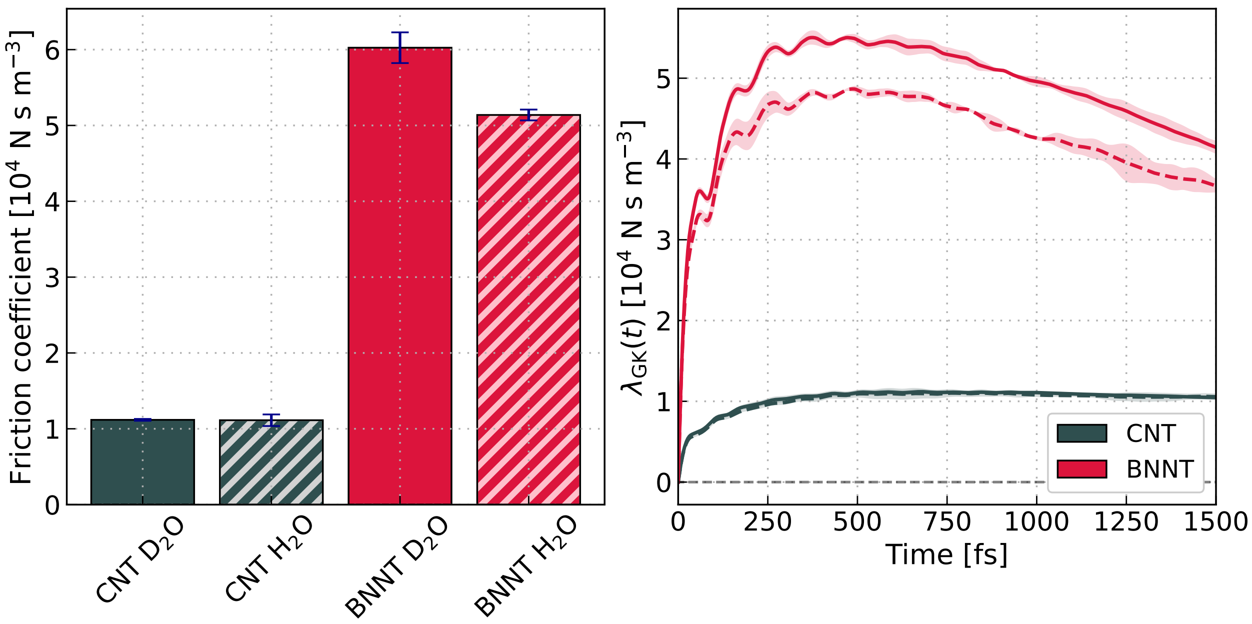

Figure S8: Dependence of the friction coefficient on the hydrogen masses used. On the left, we show the extracted friction coefficients based on the approach presented in reference [26] for the smallest CNT and BNNT with varying hydrogen mass. In the right panel, we show the established Green-Kubo relation where friction coefficient is proportional to integrated autocorrelation function. The errorbars and shaded areas around the curves correspond to the statistical error obtained by splitting the trajectory into two blocks.

DFT level predicts a similar friction coefficient of water inside the CNT while the values for the BNNT deviate almost by a factor of two with the hybrid predicting the higher friction. To understand the origin behind these trends we take a closer look at the autocorrelation function of the summed force in axial (z) direction shown in the center panel of figure S9. While the curves look almost identical for the CNT, for the BNNT we observe that the autocorrelation function is shifted by a constant value while the general trends and oscillations are very similar. This shift can be traced back to the larger average squared summed force $\langle F_z^2 \rangle$ for the hybrid in case of BNNTs. This is also supported by the friction coefficient following the established Green-Kubo relation [22, 23] based on the integrated autocorrelation shown in the right panel of figure S9. A reason for this difference in the root mean square force might stem from the generally observed increase of the bandgap going from a GGA to a hybrid functional. This way, the electrostatic interactions between the nitrogen and hydrogen are enhanced binding the water molecule stronger to the surface during the docking events and, thus, leading to a larger friction.

S2.F Nuclear quantum effects

Due to the low mass of the protons in water it is worth investigating the impact of nuclear quantum effects (NQEs) on the friction coefficient. In figure S10 we compare the values for the smallest nanotubes (12,12) computed with MD with classical nuclei and T-RPMD which includes the quantum nature of the nuclei. Due to the high computational cost associated with PIMD, the number of axial repetitions was reduced to 6 instead of 12 for all simulations to enable a fair comparison. In section S2.B we showed that for the smallest tubes the numerical values change only to a small extend. Overall, NQEs have an almost negligible impact on the friction coefficient with the MD and T-RPMD estimates agreeing with each other within error bars.

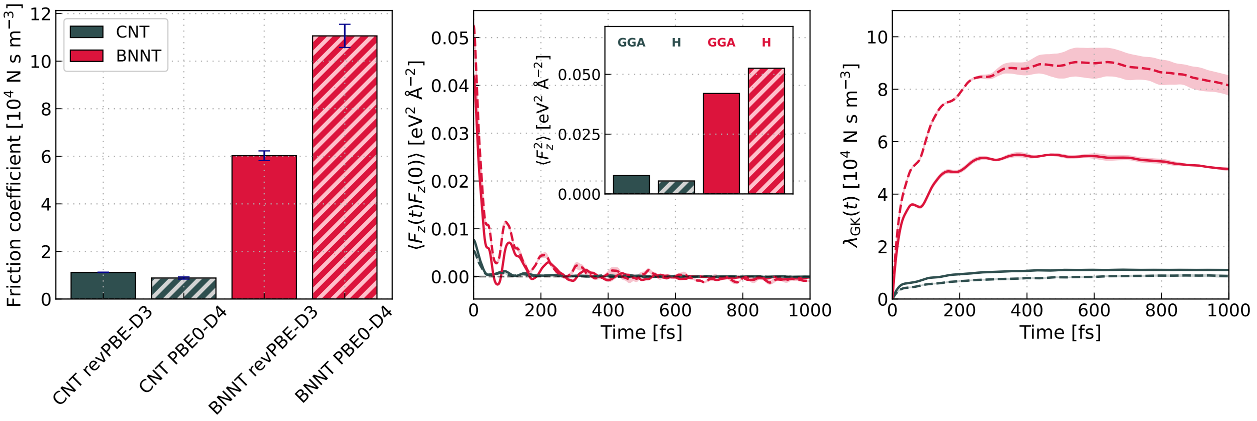

Figure S9: Dependence of the friction coefficient on the DFT functional. On the left the extracted friction coefficients based on the approach presented in reference [26] are shown. The center panel shows the autocorrelation function of the summed force in axial-direction (z) as a function of time for the smallest CNT and BNNT. Dependent on the level of theory of the simulation, the lines are either solid or dashed corresponding to GGA and hybrid (H) DFT. The inset shows a bar plot of the ensemble average of the squared summed force. In the right panel, we show the estabilshed Green-Kubo relation where friction coefficient is proportional to integrated autocorrelation function. The errorbars and shaded areas around the curves correspond to the statistical error obtained by splitting the trajectory into two blocks.

S2.G Tube flexibility

It is well known that the coupling between the phonon modes of the confining material and the water vibrations strongly affects the fluid transport across low-dimensional materials [39, 40]. Treating the confining solid as flexible and allowing the atoms to fluctuate has, thus, a crucial impact on the predicted water flux. A previous study based on classical force fields has indeed shown that water experiences a significantly lower friction in flexible compared to rigid CNTs [41]. To get a "fair" comparison to simulation studies treating the nanotubes [29] or graphene and hBN sheet [42] as rigid, here we compute the friction coefficient for rigid nanotubes. Similar to the other convergence checks, we limit this analysis to the smallest CNT and BNNT (12,12). When simulating the nanotubes as rigid bodies, we ensured that the error estimate provided by the committee disagreement in the C-NNP formalism remains sufficiently small underlying that the MLP provides a reliable estimate of the friction coefficient.

The summary of this analysis is shown in the left panel of figure S11. In line with previous simulations 41 and theory [40], we observe for both materials a $\approx 4-5$ times larger friction when the solid is treated as rigid. We also compare the summed force autocorrelation function in the left panel of S11. In contrast to our findings for the functional dependence in section S2.E, the static contribution to the friction, $\langle F_z^2\rangle$, is almost independent of the flexibility of the tube. Conversely, the oscillations of the autocorrelation function vary greatly indicating that the coupling between fluid and phonons of the solid leads to a strong decrease of the relaxation time of the force acting on the liquid in axial direction. This is what leads to higher flux rates in flexible nanotubes. Finally, relating the observations of this section to the main findings in the manuscript, the difference between predictions of the flow through CNTs made by classical force fields [29] deviate even further from our ML-based simulations when we treat the walls as rigid. This highlights the importance of describing the interatomic interactions at quantum-mechanical accuracy to obtain insight into the radius and material dependence in nanotubes.

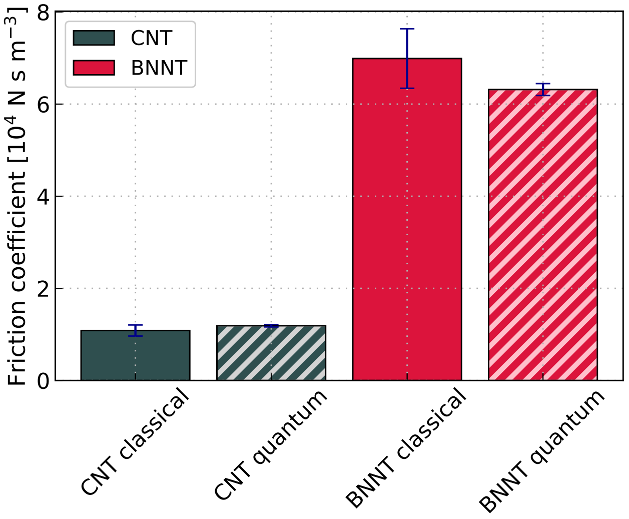

Figure S10: Impact of nuclear quantum effects on the friction coefficient. The friction coefficient were extracted from the established Green-Kubo relation using the approach presented in reference [26]. The errorbars and shaded areas around the curves correspond to the statistical error obtained by splitting the trajectory into two blocks.

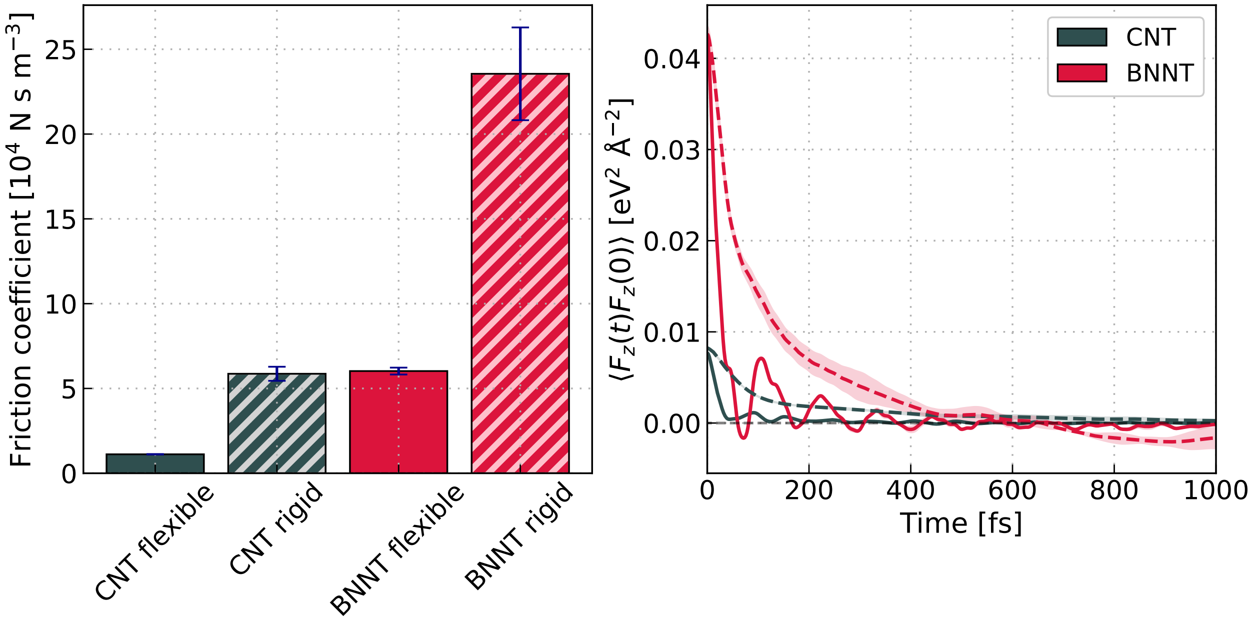

Figure S11: Impact of the flexibility of the tube walls on the friction coefficient. On the left the extracted friction coefficients based on the approach presented in reference [26] are shown. In the right panel we show the autocorrelation function of the summed force in axial-direction (z) as a function of time for the smallest CNT and BNNT. The solid lines correspond to treating the walls as flexible and represent the results presented in the manuscript. Dashed lines, conversely, illustrate the autocorrelation function in rigid nanotubes. The error bars on the left and shaded areas on the right correspond to the statistical error obtained by splitting the trajectory into two blocks.

References

[1] A. Sam, K. Vishnu Prasad, and S. P. Sathian, "Water flow in carbon nanotubes: The role of tube chirality," *Physical Chemistry Chemical Physics*, vol. 21, no. 12, pp. 6566–6573, 2019.

[2] G. Tocci, L. Joly, and A. Michaelides, "Friction of water on graphene and hexagonal boron nitride from Ab initio methods: Very different slippage despite very similar interface structures," *Nano Letters*, vol. 14, no. 12, pp. 6872–6877, 2014.

[3] T. D. Kühne, M. Iannuzzi, M. Del Ben, V. V. Rybkin, P. Seewald, F. Stein, T. Laino, R. Z. Khaliullin, O. Schütt, F. Schiffmann, D. Golze, J. Wilhelm, S. Chulkov, M. H. Bani-Hashemian, V. Weber, U. Borštnik, M. Taillefumier, A. S. Jakobovits, A. Lazzaro, H. Pabst, T. Müller, R. Schade, M. Guidon, S. Andermatt, N. Holmberg, G. K. Schenter, A. Hehn, A. Bussy, F. Belleflamme, G. Tabacchi, A. Glöß, M. Lass, I. Bethune, C. J. Mundy, C. Plessl, M. Watkins, J. VandeVondele, M. Krack, and J. Hutter, "CP2K: An electronic structure and molecular dynamics software package -Quickstep: Efficient and accurate electronic structure calculations," *Journal of Chemical Physics*, vol. 152, p. 194103, 2020.

[4] S. Goedecker and M. Teter, "Separable dual-space Gaussian pseudopotentials," *Physical Review B - Condensed Matter and Materials Physics*, vol. 54, no. 3, pp. 1703–1710, 1996.

[5] J. VandeVondele and J. Hutter, "Gaussian basis sets for accurate calculations on molecular systems in gas and condensed phases," *Journal of Chemical Physics*, vol. 127, p. 114105, 2007.

[6] J. P. Perdew, K. Burke, and M. Ernzerhof, "Generalized gradient approximation made simple," *Physical Review Letters*, vol. 77, no. 18, pp. 3865–3868, 1996.

[7] S. Grimme, J. Antony, S. Ehrlich, and H. Krieg, "A consistent and accurate ab initio parametrization of density functional dispersion correction (DFT-D) for the 94 elements H-Pu," *Journal of Chemical Physics*, vol. 132, p. 154104, 2010.

[8] S. Grimme, S. Ehrlich, and L. Goerigk, "Effect of the Damping Function in Dispersion Corrected Density Functional Theory," *Journal of Computational Chemistry*, vol. 32, p. 1456, 2011.

[9] Y. Zhang and W. Yang, "Comment on "generalized gradient approximation made simple"," *Physical Review Letters*, vol. 80, no. 4, p. 890, 1998.

[10] G. Bussi, D. Donadio, and M. Parrinello, "Canonical sampling through velocity rescaling," *Journal of Chemical Physics*, vol. 126, p. 014101, 2007.

[11] M. Rossi, M. Ceriotti, and D. E. Manolopoulos, "How to remove the spurious resonances from ring polymer molecular dynamics," *Journal of Chemical Physics*, vol. 140, no. 23, 2014.

[12] M. Ceriotti, M. Parrinello, T. E. Markland, and D. E. Manolopoulos, "Efficient stochastic thermostatting of path integral molecular dynamics," *Journal of Chemical Physics*, vol. 133, p. 124104, 2010.

[13] J. Behler and M. Parrinello, "Generalized neural-network representation of high-dimensional potential-energy surfaces," *Physical Review Letters*, vol. 98, p. 146401, 2007.

[14] J. Behler, "Four Generations of High-Dimensional Neural Network Potentials," *Chemical Reviews*, vol. 121, pp. 10037–10072, 2021.

[15] C. Schran, K. Brezina, and O. Marsalek, "Committee neural network potentials control generalization errors and enable active learning," *Journal of Chemical Physics*, vol. 153, no. 104105, p. 104105, 2020.

[16] T. Morawietz, A. Singraber, C. Dellago, and J. Behler, "How van der waals interactions determine the unique properties of water," *Proceedings of the National Academy of Sciences of the United States of America*, vol. 113, no. 30, pp. 8368–8373, 2016.

[17] B. Cheng, E. A. Engel, J. Behler, C. Dellago, and M. Ceriotti, "Ab initio thermodynamics of liquid and solid water," *Proceedings of the National Academy of Sciences of the United States of America*, vol. 116, no. 4, pp. 1110–1115, 2019.

[18] H. Ghorbanfekr, J. Behler, and F. M. Peeters, "Insights into Water Permeation through hBN Nanocapillaries by Ab Initio Machine Learning Molecular Dynamics Simulations," *The Journal of Physical Chemistry Letters*, vol. 11, pp. 7363–7370, 2020.

[19] C. Schran, F. L. Thiemann, P. Rowe, E. A. Müller, O. Marsalek, and A. Michaelides, "Machine learning potentials for complex aqueous systems made simple," *Proceedings of the National Academy of Sciences of the United States of America*, vol. 118, no. 38, p. e2110077118, 2021.

[20] O. Marsalek and T. E. Markland, "Quantum Dynamics and Spectroscopy of Ab Initio Liquid Water: The Interplay of Nuclear and Electronic Quantum Effects," *Journal of Physical Chemistry Letters*, vol. 8, no. 7, pp. 1545–1551, 2017.

[21] I. C. Yeh and G. Hummer, "System-size dependence of diffusion coefficients and viscosities from molecular dynamics simulations with periodic boundary conditions," *Journal of Physical Chemistry B*, vol. 108, no. 40, pp. 15873–15879, 2004.

[22] L. Bocquet and J. L. Barrat, "Hydrodynamic boundary conditions, correlation functions, and Kubo relations for confined fluids," *Physical Review E*, vol. 49, no. 4, pp. 3079–3092, 1994.

[23] L. Bocquet and J. L. Barrat, "On the Green-Kubo relationship for the liquid-solid friction coefficient," *Journal of Chemical Physics*, vol. 139, no. 4, 2013.

[24] L. Bocquet, J. P. Hansen, and J. Piasecki, "Friction tensor for a pair of Brownian particles: Spurious finite-size effects and molecular dynamics estimates," *Journal of Statistical Physics*, vol. 89, no. 1-2, pp. 321–346, 1997.

[25] P. Español, J. A. De La Torre, and D. Duque-Zumajo, "Solution to the plateau problem in the Green-Kubo formula," *Physical Review E*, vol. 99, no. 2, pp. 1–6, 2019.

[26] H. Oga, Y. Yamaguchi, T. Omori, S. Merabia, and L. Joly, "Green-Kubo measurement of liquid-solid friction in finite-size systems," *Journal of Chemical Physics*, vol. 151, no. 5, 2019.

[27] J. L. Barrat and L. Bocquet, "Influence of wetting properties on hydrodynamic boundary conditions at a fluid/solid interface," *Faraday Discussions*, pp. 119–127, 1999.

[28] L. Bocquet and E. Charlaix, "Nanofluidics, from bulk to interfaces," *Chemical Society Reviews*, vol. 39, no. 3, pp. 1073–1095, 2010.

[29] K. Falk, F. Sedlmeier, L. Joly, R. R. Netz, and L. Bocquet, "Molecular origin of fast water transport in carbon nanotube membranes: Superlubricity versus curvature dependent friction," *Nano Letters*, vol. 10, no. 10, pp. 4067–4073, 2010.

[30] G. Tocci, M. Bilichenko, L. Joly, and M. Iannuzzi, "Ab initio nanofluidics: disentangling the role of the energy landscape and of density correlations on liquid/solid friction," *Nanoscale*, vol. 12, no. 20, pp. 10994–11000, 2020.

[31] P. Virtanen, R. Gommers, T. E. Oliphant, M. Haberland, T. Reddy, D. Cournapeau, E. Burovski, P. Peterson, W. Weckesser, J. Bright, S. J. van der Walt, M. Brett, J. Wilson, K. J. Millman, N. Mayorov, A. R. Nelson, E. Jones, R. Kern, E. Larson, C. J. Carey, Polat, Y. Feng, E. W. Moore, J. VanderPlas, D. Laxalde, J. Perktold, R. Cimrman, I. Henriksen, E. A. Quintero, C. R. Harris, A. M. Archibald, A. H. Ribeiro, F. Pedregosa, P. van Mulbregt, A. Vijaykumar, A. P. Bardelli, A. Rothberg, A. Hilboll, A. Kloeckner, A. Scopatz, A. Lee, A. Rokem, C. N. Woods, C. Fulton, C. Masson, C. Häggström, C. Fitzgerald, D. A. Nicholson, D. R. Hagen, D. V. Pasechnik, E. Olivetti, E. Martin, E. Wieser, F. Silva, F. Lenders, F. Wilhelm, G. Young, G. A. Price, G. L. Ingold, G. E. Allen, G. R. Lee, H. Audren, I. Probst, J. P. Dietrich, J. Silterra, J. T. Webber, J. Slavič, J. Nothman, J. Buchner, J. Kulick, J. L. Schönberger, J. V. de Miranda Cardoso, J. Reimer, J. Harrington, J. L. C. Rodríguez, J. Nunez-Iglesias, J. Kuczynski, K. Tritz, M. Thoma, M. Newville, M. Kümmerer, M. Bolingbroke, M. Tartre, M. Pak, N. J. Smith, N. Nowaczyk, N. Shebanov, O. Pavlyk, P. A. Brodtkorb, P. Lee, R. T. McGibbon, R. Feldbauer, S. Lewis, S. Tygier, S. Sievert, S. Vigna, S. Peterson, S. More, T. Pudlik, T. Oshima, T. J. Pingel, T. P. Robitaille, T. Spura, T. R. Jones, T. Cera, T. Leslie, T. Zito, T. Krauss, U. Upadhyay, Y. O. Halchenko, and Y. Vázquez-Baeza, "SciPy 1.0: fundamental algorithms for scientific computing in Python," *Nature Methods*, vol. 17, no. 3, pp. 261–272, 2020.

[32] K. Falk, F. Sedlmeier, L. Joly, R. R. Netz, and L. Bocquet, "Ultralow liquid/solid friction in carbon nanotubes: Comprehensive theory for alcohols, alkanes, OMCTS, and water," *Langmuir*, vol. 28, no. 40, pp. 14261–14272, 2012.

[33] J. P. Perdew, M. Ernzerhof, and K. Burke, "Rationale for mixing exact exchange with density functional approximations," *Journal of Chemical Physics*, vol. 105, no. 22, pp. 9982–9985, 1996.

[34] C. Adamo, M. Cossi, G. Scalmani, and V. Barone, "Accurate static polarizabilities by density functional theory: Assessment of the PBE0 model," *Chemical Physics Letters*, vol. 307, no. 3-4, pp. 265–271, 1999.

[35] E. Caldeweyher, C. Bannwarth, and S. Grimme, "Extension of the D3 dispersion coefficient model," *Journal of Chemical Physics*, vol. 147, no. 3, 2017.

[36] E. Caldeweyher, S. Ehlert, A. Hansen, H. Neugebauer, S. Spicher, C. Bannwarth, and S. Grimme, "A generally applicable atomic-charge dependent London dispersion correction," *Journal of Chemical Physics*, vol. 150, no. 15, 2019.

[37] E. Caldeweyher, J. M. Mewes, S. Ehlert, and S. Grimme, "Extension and evaluation of the D4 London-dispersion model for periodic systems," *Physical Chemistry Chemical Physics*, vol. 22, no. 16, pp. 8499–8512, 2020.

[38] M. J. Gillan, D. Alfè, and A. Michaelides, "Perspective: How good is DFT for water?," *Journal of Chemical Physics*, vol. 144, no. 13, 2016.

[39] M. Ma, F. Grey, L. Shen, M. Urbakh, S. Wu, J. Z. Liu, Y. Liu, and Q. Zheng, "Water transport inside carbon nanotubes mediated by phonon-induced oscillating friction," *Nature Nanotechnology*, vol. 10, no. 8, pp. 692–695, 2015.

[40] S. Marbach, D. S. Dean, and L. Bocquet, "Transport and dispersion across wiggling nanopores," *Nature Physics*, vol. 14, no. 11, pp. 1108–1113, 2018.

[41] A. Sam, S. K. Kannam, R. Hartkamp, and S. P. Sathian, "Water flow in carbon nanotubes: The effect of tube flexibility and thermostat," *Journal of Chemical Physics*, vol. 146, no. 23, 2017.

[42] A. R. Poggioli and D. T. Limmer, "Distinct Chemistries Explain Decoupling of Slip and Wettability in Atomically Smooth Aqueous Interfaces," *The Journal of Physical Chemistry Letters*, vol. 12, pp. 9060–9067, 2021.
